# Water will Find Its Way: Transport through Narrow Tunnels in Hydrolases

**DOI:** 10.1021/acs.jcim.4c00094

**Published:** 2024-04-26

**Authors:** Carlos Sequeiros-Borja, Bartlomiej Surpeta, Aravind Selvaram Thirunavukarasu, Cedrix J. Dongmo Foumthuim, Igor Marchlewski, Jan Brezovsky

**Affiliations:** † International Institute of Molecular and Cell Biology, Warsaw 02-109, Poland; ‡ Laboratory of Biomolecular Interactions and Transport, Department of Gene Expression, Institute of Molecular Biology and Biotechnology, Faculty of Biology, Adam Mickiewicz University, Poznań 61-614, Poland; § National Institute of Nuclear Physics (INFN), Sezione di Roma Tor Vergata, Rome 00133, Italy

## Abstract

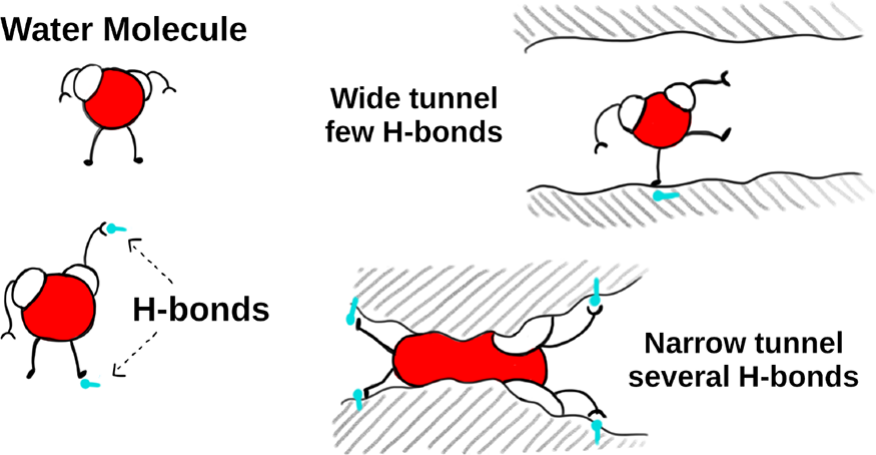

An aqueous environment is vital for life as we know it, and water is essential for nearly all biochemical processes at the molecular level. Proteins utilize water molecules in various ways. Consequently, proteins must transport water molecules across their internal network of tunnels to reach the desired action sites, either within them or by functioning as molecular pipes to control cellular osmotic pressure. Despite water playing a crucial role in enzymatic activity and stability, its transport has been largely overlooked, with studies primarily focusing on water transport across membrane proteins. The transport of molecules through a protein’s tunnel network is challenging to study experimentally, making molecular dynamics simulations the most popular approach for investigating such events. In this study, we focused on the transport of water molecules across three different α/β-hydrolases: haloalkane dehalogenase, epoxide hydrolase, and lipase. Using a 5 μs adaptive simulation per system, we observed that only a few tunnels were responsible for the majority of water transport in dehalogenase, in contrast to a higher diversity of tunnels in other enzymes. Interestingly, water molecules could traverse narrow tunnels with subangstrom bottlenecks, which is surprising given the commonly accepted water molecule radius of 1.4 Å. Our analysis of the transport events in such narrow tunnels revealed a markedly increased number of hydrogen bonds formed between the water molecules and protein, likely compensating for the steric penalty of the process. Overall, these commonly disregarded narrow tunnels accounted for ∼20% of the total water transport observed, emphasizing the need to surpass the standard geometrical limits on the functional tunnels to properly account for the relevant transport processes. Finally, we demonstrated how the obtained insights could be applied to explain the differences in a mutant of the human soluble epoxide hydrolase associated with a higher incidence of ischemic stroke.

## Introduction

An aqueous environment is essential for life as we know it, and water is a prerequisite for nearly all biochemical processes at the molecular level. Water can influence the folding of proteins,^1−3^ their dynamics,^4^ and actively participate in chemical transformations.^5^ Proteins can utilize water molecules in various ways: as proton donors or receivers,^6^ molecular stabilizers,^7^ molecular lubricants enhancing protein dynamics,^8^ and as significant contributors behind the enthalpy–entropy compensation in protein–ligand binding.^9^ In this regard, proteins must transport water molecules across their internal network of tunnels to reach the desired action sites, either within them or as molecular pipes to control the cellular osmotic pressure.

The transport of molecules through a protein’s tunnel network has proven challenging to study experimentally, with a few exceptions.^10^ Therefore, the most suitable approach to study such events is molecular dynamics (MD) simulations, a methodology with its own limitations. Nevertheless, the principal advantage of MD simulations is that it provides a detailed atomic picture of the transport process itself, the tunnel pathways employed by the molecules, and even approximated transport rates.^11−13^ Even though water plays a crucial role in enzymatic activity and stability, its transport has been somewhat neglected, with studies mainly focused on water transport across membrane proteins like aquaporins.^14−17^

Water transport at a molecular level has been extensively studied in the nanomaterials field, where researchers have tested the peculiarities and limits of water transport through various molecular barriers.^18−21^ On the biological sciences, the focus of water transport in proteins is primarily on the movement across membranes facilitated by specialized transmembrane proteins, such as aquaporins. Within these proteins, the so-called single-file transport of water can be regarded as the spatial limit required for transport.^13,22,23^ Here, water molecules are arranged in a single lane and move through tunnels at least one water molecule wide, with a commonly accepted radius of 1.4 Å.^24−26^

Recent research has revealed that the methods for assessing a protein’s tunnel network and the migration of small molecules do not consistently produce equivalent results. Some water molecules could not be traced to a defined tunnel, indicating either the method’s limitations in detecting tunnels or the possibility that water molecules could traverse the protein through inconspicuous empty spaces.^27^ In this paper, to reconcile these observations, we explore the hypothesis that water transport in proteins can occur through considerably narrow tunnels with a bottleneck radius below 1.4 Å and that this contribution is not negligible. As model systems, we focus on the tunnel network across three members of the α/β-hydrolase superfamily, emphasizing the tunnels used for water transport. Following this, we characterize these narrow tunnels and their interactions with water molecules. We demonstrate that water molecules can overcome van der Waals repulsion by increasing the number of hydrogen bonds. Next, to showcase the utility of the insights obtained, we analyze two variants of human epoxide hydrolase, a wild type and a mutant, resulting from a single nucleotide polymorphism (E470G) associated with an increased risk of ischemic stroke.^28^ We show that these narrow tunnels can identify structural differences in protein variants that are not easily discernible with traditional analyses. Finally, we suggest revisiting the current considerations of the parameters of functional tunnels.

## Materials and Methods

### Preparation of Structures

The crystallographic structures for the systems were obtained from the Protein Data Bank^29^ (PDB): haloalkane dehalogenase (Hal) from *Rhodococcus rhodochrous*(30) (PDB: 4E46), epoxide hydrolase I (Epx) from *Solanum tuberosum*(31) (PDB: 2CJP), open^32^ and closed^33^ states of lipase (Lip) from *Diutina rugosa* (previously *Candida rugosa*) (PDB: 1CRL and 1TRH, respectively), and human epoxide hydrolase^34^ (hEpx) (PDB: 4X6X). In the case of Epx, two chains corresponding to two distinct enzyme structures were present in the asymmetric units of the respective PDB record. To provide more conformational diversity, both structures underwent follow-up modeling after synchronizing their composition by removing residue K2 present only in chain A. To obtain the E470G variant of hEpx, residue E470 was renamed glycine, and the side-chain atoms were removed from hEpx. All structures were processed by eliminating all nonprotein molecules except the crystallographic waters.

The initial protonation states were determined with the H++ 3.0 Web server^35^ at pH 8.5^36^ for Hal, 6.8^37^ for Epx, 8.0^38^ for Lip, and 7.4^39^ for hEpx and E470G, reflecting respective catalytic pH optima for Hal, Epx, and Lip, whereas for hEpx and E470G, the physiological pH was used. For the Lip system, residue E208 was differentially protonated for the open and closed states; therefore, to build a uniform topology, this residue was manually deprotonated in both conformations. For all proteins, water molecules were initially placed around the solute using a tandem approach based on the 3D reference interaction site model theory^40^ (3D-RISM) and the Placevent^41^ algorithm. All predicted 3D-RISM waters were subsequently combined with crystallographic waters, keeping only the water molecules at least 2 Å away from the protein using EDIAscorer,^42^ a method to compute electron density for individual atoms in a crystal structure. The resulting system was then processed with the tleap module of the Amber18^43^ package. Each protein was positioned at the center of a periodic truncated octahedral box, at a distance of 10 Å away from the edges, and solvated with the three-charge, four-point rigid OPC water model.^44^ Na^+^ and Cl^–^ ions were initially added to neutralize the system’s charge and subsequently to reach a salt concentration of 0.1 M. The MD simulations were performed with the pmemd.cuda module of Amber18,^45,46^ employing the ff14SB^47^ force field. Finally, the hydrogen mass repartitioning (HMR) method^48^ was applied to the solute to enable a simulation time step of 4 fs.

### MD Protocol

The systems underwent five minimization cycles, with a stepwise release of positional restraints on the protein atoms. The minimization cycles comprised 100 steps of the steepest descent minimization algorithm followed by 400 steps of the conjugated gradient. In the initial minimization cycle, harmonic positional restraints were imposed on all heavy atoms of the protein with a force constant of 500 kcal·mol^–1^·Å^–2^. Subsequent cycles applied restrictions only to the backbone atoms with force constants of 500, 125, 25, and 0.0001 kcal·mol^–1^·Å^–2^ sequentially. Following the minimization workflow, a brief heating round of NVT MD simulation was applied while keeping the protein’s heavy atoms restrained with a force constant of 5 kcal·mol^–1^·Å^–2^. The heating cycle involved 20 ps of MD simulation from 0 to 200 K using the Langevin thermostat with a collision frequency of 2 ps^–1^, a coupling constant of 1 ps, and a simulation time step of 4 fs. The long-range electrostatic interactions were computed using the particle mesh Ewald summation,^49,50^ and all bonds involving hydrogen atoms were constrained using the SHAKE^51^ algorithm.

Subsequently, four equilibration cycles were conducted. First, in the NVT simulation, the temperature was raised to the target value of 310 K in 100 ps by employing the same parameters as previously described. The temperature was maintained constant for 900 ps, and during this process, harmonic positional restraints were applied to the protein’s heavy atoms with a force constant of 5 kcal·mol^–1^·Å^–2^. Next, 1 ns of NPT MD simulation was run, with pressure controlled using the weak-coupling Berendsen barostat with a coupling constant of 1 ps, and positional restraints were applied only to the backbone atoms using the same force constant as in the previous stage followed by 1 ns of NPT simulation without positional restraints. Finally, 200 ns of NPT simulation was performed to enable equilibration of internal water molecules, using the same settings as before except for using Monte Carlo barostat. The final snapshots from simulations of all variants of each system were utilized as their initial seeding structures for adaptive MD simulations.

The production stage utilized the High-Throughput Molecular Dynamics^52^ package (HTMD v1.13.10) with Amber18 as the MD simulation engine. For HTMD settings, five parallel MD simulation runs per epoch were configured with 10 epochs in total. The HTMD package facilitates the construction of Markov state models (MSM) after each epoch, prioritizing less-sampled conformational states in subsequent rounds to enhance system sampling.^52−54^ To build the MSM, the dihedral angles of some selected residues were employed, as described hereafter. Initially, the geometric center of the active site residues for each system was calculated. Residues N41, D106, W107, E130, and H272 for Hal; D105, Y154, Y234, D265, and H300 for Epx; G123, G124, S209, A210, E341, and H449 for Lip; and D335, Y383, Y466, and H524 for hEpx and E470G were used as centers of selection spheres (residue numbers follow their PDB structures). The dihedral angles of all residues within a sphere of 12 Å radius for Hal, Epx, hEpx, and E470G, and 18 Å radius for Lip were used as metrics for MSM building, with a TICA^55^ dimension of 3 and a lag time of 2.

The equilibration phase in each HTMD epoch was composed of two short NVT and NPT MD simulations of 250 ps each. During these rounds, the systems were heated from 0 to 310 K with a Langevin thermostat and harmonic positional restraints applied to the backbone atoms with a force constant of 5 kcal·mol^–1^·Å^–2^. Finally, a 100 ns unrestrained NVT production simulation was conducted, employing the Berendsen thermostat to conserve the momenta of individual atoms^56^ and a saving frequency of 10 ps as only the NVT production simulations were fully supported by HTMD package.

Three additional runs of adaptive sampling simulations of Hal systems were performed to evaluate the dependence of water transport on the simulations setup: (i) simulations with 2 fs time step and without HMR applied, (ii) simulations at a lower temperature of 300 K, and (iii) simulations using the TIP3P model^57^ for water molecules. All remaining settings in these three runs were kept as described above.

### Tunnel Network and Water Transport Analyses

The trajectories underwent analysis using the cpptraj^58^ module of Amber18, CAVER^59^ v3.02, AQUA-DUCT^60^ v1.0.11, TransportTools^61^ v0.9.0, and Python3 *in-house* scripts. For the tunnel network calculations with CAVER, the divide-and-conquer approach^62^ was applied, setting the probe radius to 0.7 Å and the shell radius to 3.0 Å for Hal, Epx, hEpx, and E470G and 5.0 Å for Lip. CAVER starting atoms for the tunnel search were chosen as follows: D106-CG, W107-CD2, F168–N, and L246-N for Hal; W106-CD2, L181-N, Y235-CB, and D265-CA for Epx; G123-O, E126-CG, G342-CA, and T416-HA for Lip; and Y466-CD1 and H524-CA for hEpx and E470G. The remaining parameters were left as default. These atoms were selected based on their center of geometry, presenting one of the lowest root-mean-square fluctuations (RMSF) and being inside the deepest part of the cavities described in the literature for each protein.^63−72^ To achieve consistent tunnel entrances, HDBSCAN v0.8.27^73^ was used at the tunnel end points for clustering, with the following parameters: *cluster_selection_epsilon* of 1.5, *allow_single_cluster* set to True, and *min_samples* and *min_cluster_size* set to 5. The formed clusters were considered independent tunnels, and the tunnels identified as noise were discarded. For water tracking analyses, the same atoms described earlier were used for the object definition in AQUA-DUCT, employing a sphere of 6.0 Å for Hal and 4.0 Å for Epx, Lip, hEpx, and E470G. The remaining parameters were set to default. In TransportTools, the average-link hierarchical clustering method with a threshold of 1.0 Å was applied, utilizing the exact matching analysis for the assignment of transport events. All other settings were maintained as a default.

**Figure 1:**
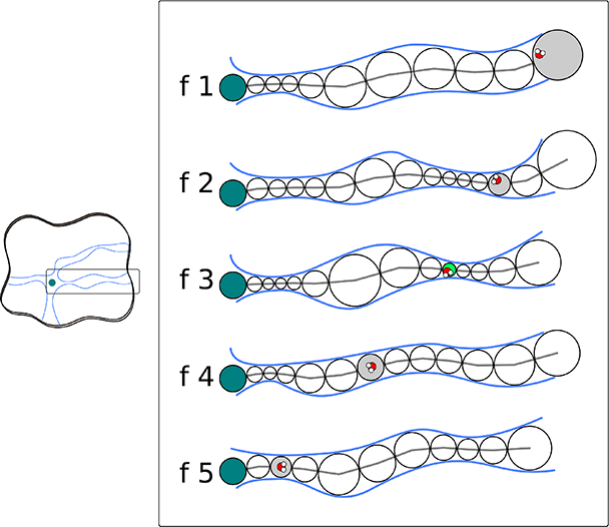
Water transport event and minimal event sphere definition. A protein can contain several tunnels and transport events (left), and in one tunnel, a transport event is composed of several frames (enclosed right). In each frame of a transport event, a tunnel is present as a set of tunnel spheres (empty circles) along a centerline (gray line) that joins the active site (turquoise circles) with the exterior. In each frame, the migrating water molecules are assigned to a single event sphere of the tunnel (gray circles), and among all of them, the smallest corresponds to the minimal event sphere (green circle).

For each water transport event assigned by TransportTools to a defined tunnel for at least 70% of its duration, a postprocessing step was performed to obtain more detailed information about the nature of the transport. Here, a tunnel is regarded as a collection of tunnel spheres along a central line, and in each frame of a transport event, the water molecule involved is assigned to the closest tunnel sphere (Figure 1). Following this concept, a transport event comprises a collection of event spheres to which a migrating water molecule was assigned, with a restriction of a single event sphere per frame. Our subsequent analyses focused on frames where the water molecule was assigned to the smallest sphere along the event, termed the minimal event sphere (Figure 1). For these selected frames, the radius of the minimal event sphere and the hydrogen bonds (H-bonds) between the water molecule and protein were considered. Energetically significant H-bonds with a distance between donor and acceptor heavy atoms up to 3.5 Å and the angle between the acceptor heavy atom, the hydrogen, and the donor heavy atom in the range 135–180° were identified using the cpptraj program.^74^

## Results and Discussion

### Tunnel Networks Identified in Hal, Epx, and Lip from Adaptive MD Simulations

The MD simulations for all of the proteins exhibited stable behavior during the 5 μs of simulation, with root-mean-square deviation (RMSD) averages of 1, 2, and 2.5 Å for Hal, Epx, and Lip, respectively (Figures S1–S3). Although these differences in RMSD might raise some concerns about the stability of the systems, the RMSF analyses showed that this is not the case. The size variations in proteins are different, and in Hal, the protein is densely packed with fluctuations mainly occurring at the N- and C-terminal regions (Figure S4). Similarly, in Epx and Lip, some regions show higher flexibility than the rest of the system (Figures S5 and S6), corresponding to areas with large b-factors in the crystals, indicating high flexibility.

TransportTools results for Hal showed the presence of all the tunnels described in the literature,^63^ namely, *p1*, *p2a*, *p2b*, *p2c*, and *p3*(64) (Figure S7a,b and Table S1), with all of them used for water transport to some extent, although the *p1* tunnel can be considered as the main water conduit. The results also revealed new tunnels for water transport: a top tunnel near *p1* (called the *up* tunnel here), located closer to helix α5′ (Figure S7c,d – salmon color), and a side tunnel perpendicular to the *p1* tunnel, opposite to the *p2* tunnel (called the *side* tunnel here), between the α/β and cap domains (Figure S7c,d light green color). For Epx, the three main tunnels described by Mitusińska et al. are present,^65^ but tunnel *TM1* is formed by two TransportTools superclusters (Figure S8a,b and Table S1). The results also show numerous new tunnels that transport water, albeit in smaller quantities, which may correspond to the outliers observed by Mitusińska et al. (Figures S8c,d and S9). Lip results showed predominant water transport in the *main* tunnel described in previous studies^66−69^ (Figure S10a,b and Table S1). Additionally, several important tunnels for water transport were identified, most in the same region of the main tunnel (Figure S10c,d) and fewer in different regions (Figure S11). The results also revealed the presence of the hypothesized *ester* exit tunnel;^70^ however, only two water molecules were transported via this tunnel (Figure S10a,b and Table S1). Tunnel network analysis for the three systems showed the presence of all tunnels described in the literature with several new tunnels found, mainly for Epx and Lip. Regarding the difference in water transport among the three investigated enzymes (Table S2), the least water molecules were transported by Hal, which has only a single permanently available tunnel *p1* capable of significant opening to 3.0 Å and facilitating transport of 2000 water molecules (Table S1). In Epx, the permanent *TM1* tunnel is about twice as wide as the *p1* tunnel, allowing for transporting almost 50,000 water molecules. Additionally, there are two other tunnels with parameters similar to the *p1* tunnel, i.e., *TC/M* and *TM2*, each allowing the transport of circa 2000 water molecules and two auxiliary tunnels (Table S1). Finally, the *main* tunnel of Lip has comparable properties to *the TM1* tunnel of Epx, enabling the transport of over 30,000 water molecules. The *main* tunnel was supported by a network of four other tunnels with considerable water transport capacity similar to that of the *p1* tunnel of Hal and six auxiliary tunnels (Table S1). Altogether, these tunnels provided Lip with an overall water transport capability comparable to Epx (Table S2).

### Water Transport within the Tunnel Networks of Hal, Epx, and Lip

Overall, we collected 2222, 17,786, and 11,086 water transport events that could be unambiguously assigned to tunnels in Hal, Epx, and Lip, respectively (Table S2), constituting 23–84% of all transport events analyzed. A limited number of events (<3%) could not be assigned even to the overall superclusters generated by TransportTools across all 50 simulations of each hydrolase. However, the main reason for misalignment between water events and tunnels originated from attempts to match these events exactly to particular tunnels found by CAVER in the trajectory part where the transport event occurred, which is in line with recent observations for eight soluble epoxide hydrolases.^27^ In general, mismatches stem from the approximation of asymmetric internal voids by spherical tunnels produced by CAVER,^75^ promoted by maintaining only the tunnel with the best throughput for the tractability of tunnel clustering in each frame,^76^ restricting the description of the entire tunnel geometry.

**Figure 2:**
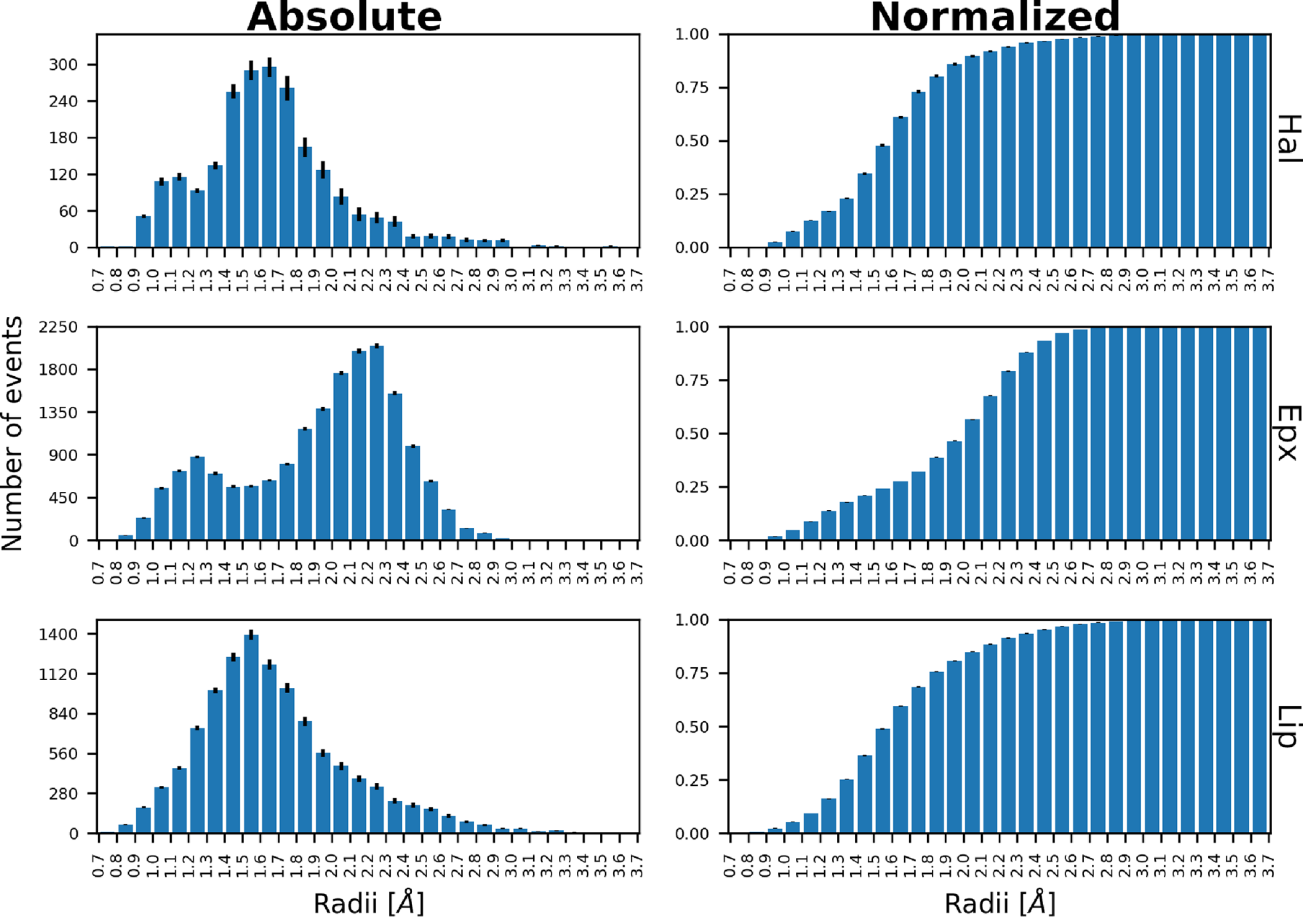
Distribution of *minimal event sphere radii* for water transport in Hal, Epx, and Lip. The absolute number of transport events and their corresponding normalized cumulative distribution are shown. The variances were estimated by resampling 100 times 45 of 50 available MD simulations; the number of simulations contributing to each bin is shown in Figure S13.

For the assigned events, the distribution of *minimal event sphere* radii for Hal exhibited two peaks, with the major peak at 1.6–1.7 Å and a minor peak at 1.1–1.2 Å (Figure 2). A similar trend was observed for Epx, with a minor peak at 1.2–1.3 Å and a major peak at 2.2–2.3 Å (Figure 2). However, this trend changed in Lip, where the curve showed a clear single peak at 1.5 and 1.6 Å (Figure 2). An examination of these distributions by the tunnels involved revealed that the bimodal nature of Epx transport was primarily due to two tunnels (Figure S12). A somewhat similar phenomenon was observed for Hal, in which two tunnels formed a smaller peak at around 1.0–1.2 Å (Figure S12). This bimodal behavior was not the case in Lip because all tunnels featured a peak at 1.5 and 1.6 Å (Figure S12). Cumulative distribution results displayed sigmoidal behavior for all systems, although Epx deviated slightly due to its stronger bimodal nature (Figure 2). Given 1.4 Å as the widely accepted radius for a water molecule,^24−26^ the number of transport events below this threshold was 22.7, 17.7, and 25.0% for Hal, Epx, and Lip, respectively (Figure 2), not a negligible quantity at all.

Before we considered the implications of these observations, we had tested that such narrow *minimal event sphere* radii were not due to well-known limitations arising from the spherical approximation of transport tunnels by CAVER.^75^ Such an approximation could overlook available voids in the protein structure adjacent to the tunnel sphere that could easily accommodate the migrating water molecule. By measuring the surface–surface distances between water molecules in the *minimal event sphere* and the closest protein atoms (Figure S14), we verified that water molecules were indeed passing through restricted empty spaces, marked by frequent atomic overlaps for most of these events.

Furthermore, we have excluded the possibility that these passages constitute artifacts from the use of a 4 fs time step without HMR applied to the solvent.^48^ The MD simulations of Hal at a 2 fs time step without HMR exhibited comparable behavior in RMSD and RMSF to the previous MD simulations with the longer time step and HMR applied (Figures S15 and S16). Importantly, we have observed a similar distribution of water migration events according to the *minimal event sphere* radii (Figure 2 and Figure S17a) despite likely divergent sampling of tunnel conformations between these two adaptive sampling simulation runs. The agreement further extended to the amount of water molecules (21.8%) migrating through tunnels with a radius below 1.4 Å in the simulations with a 2 fs time step, comparable to 22.7% observed with the initial simulation settings.

Finally, we explored the sensitivity of these observations to the different water model (TIP3P versus OPC) and temperatures (300 versus 310 K) employed during the MD simulations of the Hal enzyme. As expected, simulations in TIP3P sampled more pronounced movements of the Hal protein (Figures S19 and S20), in line with the overestimated mobility of TIP3P water molecules.^77,78^ Conversely, these movements were more restricted at lower temperatures (Figures S21 and S22). In TIP3P, the overall transport of water molecules via Hal tunnels was markedly enhanced at all *minimal event sphere* radii (Figure S17b), including an almost 3-fold increase in the amount of water molecules transported through tunnels with a radius below 1.4 Å (1351 molecules in TIP3P versus 505 molecules in OPC). However, major increases were observed for wider tunnel geometries, which aligns with a recent study exposing the systematic observation of faster and more parallel transport of TIP3P over OPC water molecules through tunnels of three different enzymes.^79^ At the lower temperature, the overall number of migrating water molecules was comparable to the simulations at 310 K but the narrow tunnels of Hal were utilized by water molecules with approximately halved frequency (Figure S17c), which could be due to their insufficient kinetic energy to overcome the energy barriers along the path, break from the stabilizing H-bonds, and less frequent opening of the tunnels.

For proteins specialized in water transport, such as aquaporins, the accepted minimum radius of a functional state is the size of a water molecule, leading to the so-called single-file water transport.^22^ Given the results obtained for the three hydrolases studied here, it becomes evident that water can often be transported below the limits recognized for such optimized proteins, where the optimized flow of water molecules is critical to maintaining cellular function. Intrigued by these results, we focused on unraveling the characteristics and limits of water transport through enzyme tunnels with radii below 1.4 Å.

### Water Transport through Narrow Tunnels

**Figure 3:**
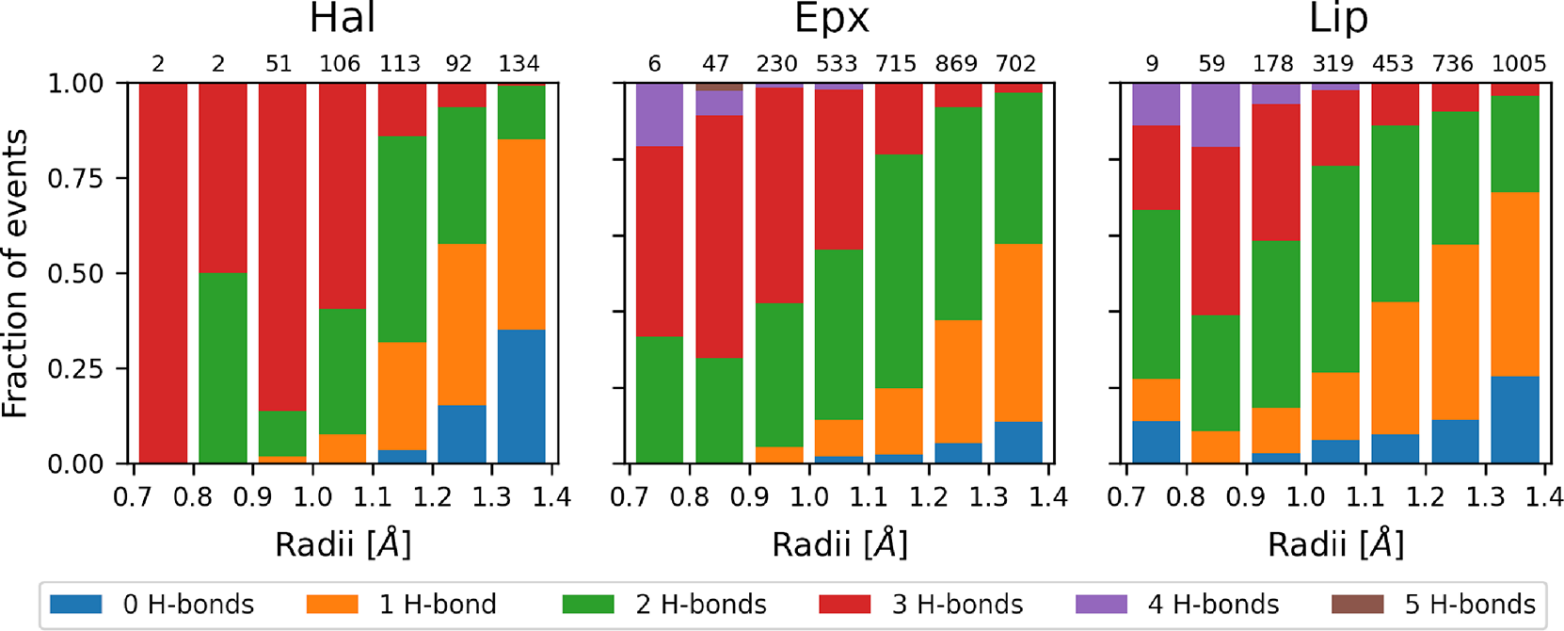
Hydrogen bond distribution of water events at narrow tunnels. H-bonds formed between water and protein at narrow tunnels with bottleneck radii below 1.4 Å. The normalized distributions for Hal, Epx, and Lip are shown, and the total number of events in each bin is displayed at the top.

We examined H-bonds between the protein and water for each frame in which the water molecule was inside the *minimal event sphere*. Notably, water demonstrated a tendency to form several H-bonds with the protein at a lower *minimal event sphere* radius, a consistent pattern observed across the three systems studied here (Figure 3). This trend persisted when considering all radii for the analysis and was further emphasized by an apparent inverse correlation between the radius and the number of H-bonds (Figures S23 and S24).

**Figure 4:**
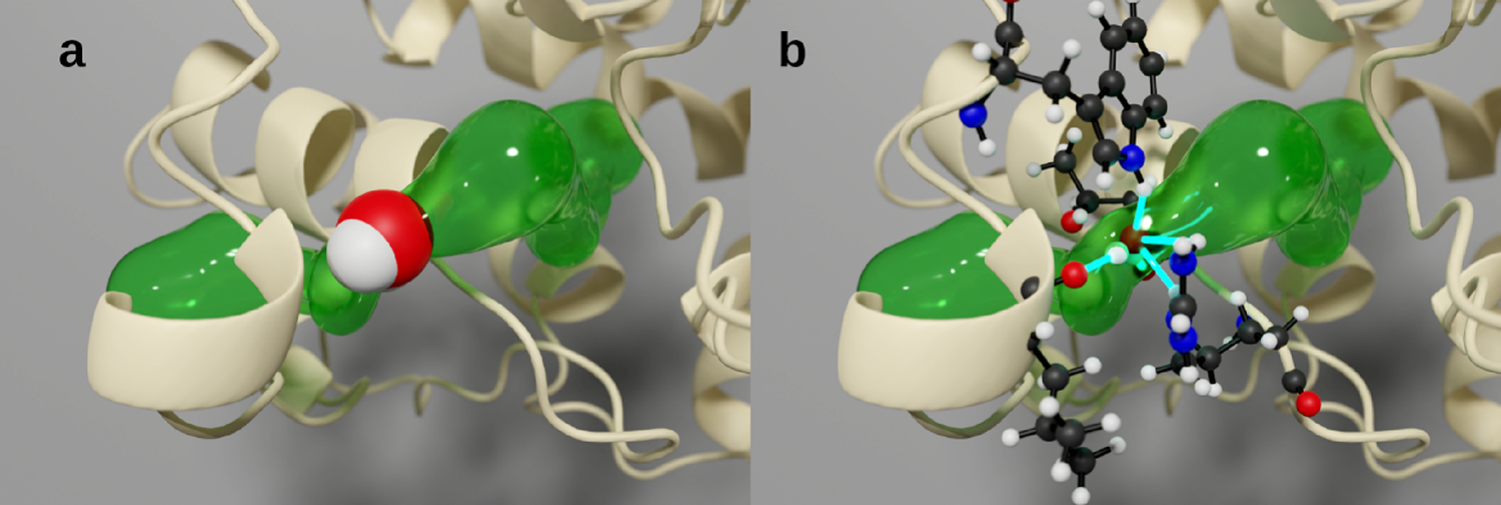
Water molecule forming hydrogen bonds while traversing a narrow tunnel. (a) Water molecule (van der Waals volume) inside Hal (beige cartoon), traversing a narrow tunnel (green surface). The volume of the water molecule exceeds the available space in the tunnel. (b) Alternative representation of the same frame, with the water molecule (depicted as ball and sticks) inside Hal (beige cartoon), forming multiple H-bonds (cyan sticks) with protein side chain and backbone atoms (ball and sticks) during its passage through a narrow tunnel (green surface).

Our hypothesis suggests that water molecules require multiple H-bonds to stabilize their transport through narrow tunnels, enabling them to overcome repulsive van der Waals forces between water and protein atoms (Figure 4). Also, we have noted weaker electrostatic interactions of protein with the water molecules in narrow tunnels of Hal compared to Epx and Lip (Figure S25), which agrees with the lower number of H-bonds stabilizing water molecules in such narrow tunnels of Hal (Figure S23). The compensation between unfavorable van der Waals and favorable H-bonding interactions observed across all investigated proteins aligns with a mutational study on the transmembrane protein 175 from *Chamaesiphon minutus*,^26^ where an increase in wetting behavior at the gate was observed despite the tunnel radius remaining constant. The mutation involved changing the hydrophobic gate residues Ile and Leu to the polar Asn. While this observation pertains to wetting rather than transport, it aligns with our findings. The outcomes of our H-bond analysis differ from the notion of single-file water transport, where the presence of H-bond donors/acceptors along the entire tunnel length is believed to increase the energy barrier for water transport.^22,23^ In the realm of nanomaterials, a pore radius of 2.85 Å in a graphdiyne membrane was considered the ultimate size limit for effective water transport.^20^ Despite being almost twice the size of a water molecule, the entirely hydrophobic nature of these artificial structures prevents water from physically passing through the open pores.^18^

**Figure 5:**
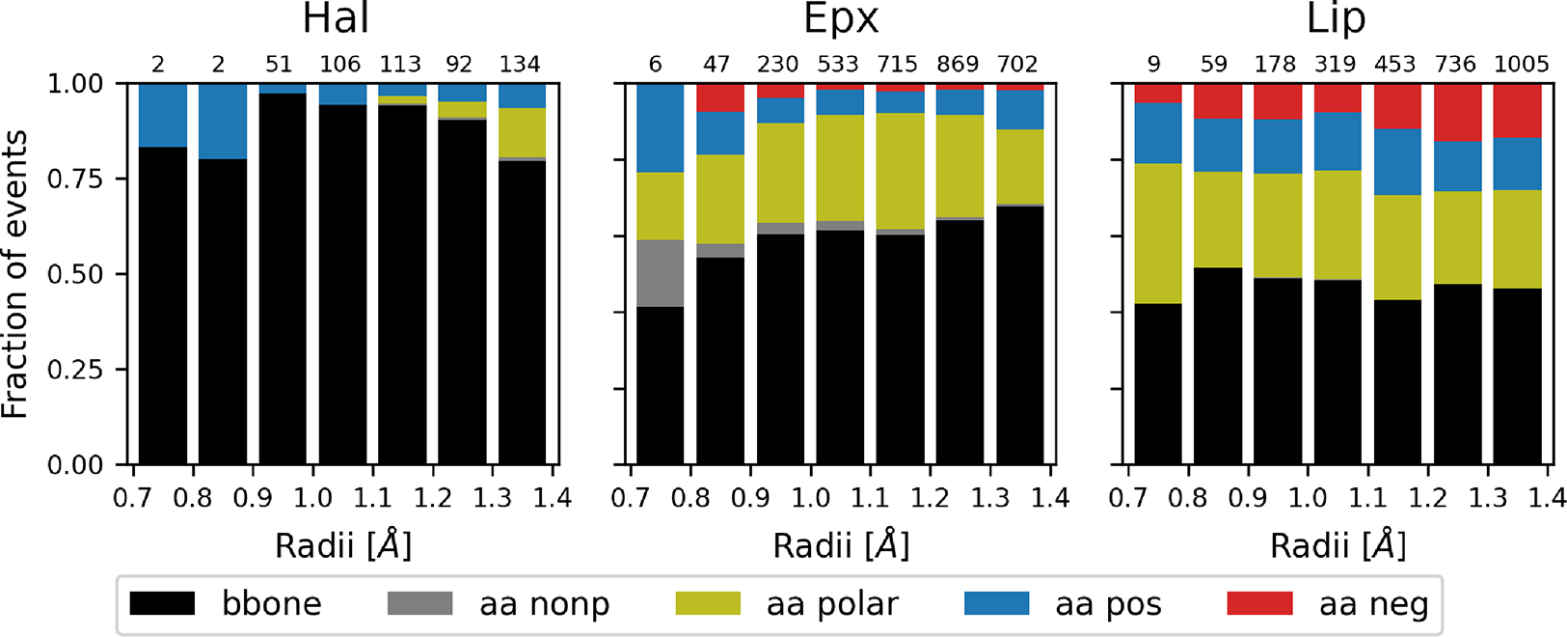
Distribution of atoms responsible for hydrogen bonding in water transport. The protein atoms involved in hydrogen bonding with water in transport events for Hal, Epx, and Lip. Side-chain atoms considered nonpolar (Ala, Gly, Ile, Leu, Met, Phe, Pro, Trp, and Val), polar (Asn, Cys, Gln, Ser, Thr, and Tyr), positive (Arg, His, and Lys), or negative (Asp and Glu). The total number of events for each bin is indicated at the top.

Furthermore, our analysis of which atoms on the residues were responsible for the H-bonding network revealed a predominance of the backbone atoms (Figure 5 and Figure S26). Given that each H-bond analyzed could involve multiple atoms making contacts, the distributions visibly changed for Hal and Epx, not only in quantity but also in the position of the peaks (Figure S26). In the Lip case, only the quantity changed, maintaining the peak almost at the same radius (Figure S26). Although there is no clear trend regarding the type of atoms forming H-bonds with water during transport across all enzymes, it is reasonable to conclude that backbone atoms play a crucial role, especially at lower radii. This insight leads to the hypothesis that water transport through considerably narrow tunnels is fold-specific and may be preserved within protein families. Supporting this observation, a recent study on epoxide hydrolases demonstrated that tunnels are conserved structural features of proteins.^80^ Although mutational studies on tunnels have indicated changes in specificity^81^ and catalytic rate,^82^ the targets were primarily the major tunnels, and the observed effects were related to substrate/product intake and release.

### Case Study: Human Epoxide Hydrolase and Risk of Ischemic Stroke

To illustrate the often overlooked significance of narrow tunnels, a comparative analysis was conducted between the human soluble epoxide hydrolase of wild type (hEpx) and the variant E470G. hEpx utilizes water as a cosubstrate for the hydrolysis of epoxides into their corresponding diols, making water a crucial component for its proper functioning.^83,84^ hEpx is involved in the hydrolysis of epoxyeicosatrienoic acids, which possess vasodilator, anti-inflammatory, analgesic, antifibrotic, and antihypertensive properties.^85^ Polymorphisms in the gene encoding hEpx have been linked to varying enzyme activity rates,^39^ thereby being associated with susceptibility or protection against incident ischemic stroke.^28^ Notably, one such variant, E470G, is correlated with an increased risk of ischemic stroke in the African-American population, although the underlying molecular mechanism remains unclear.^28^

**Figure 6:**
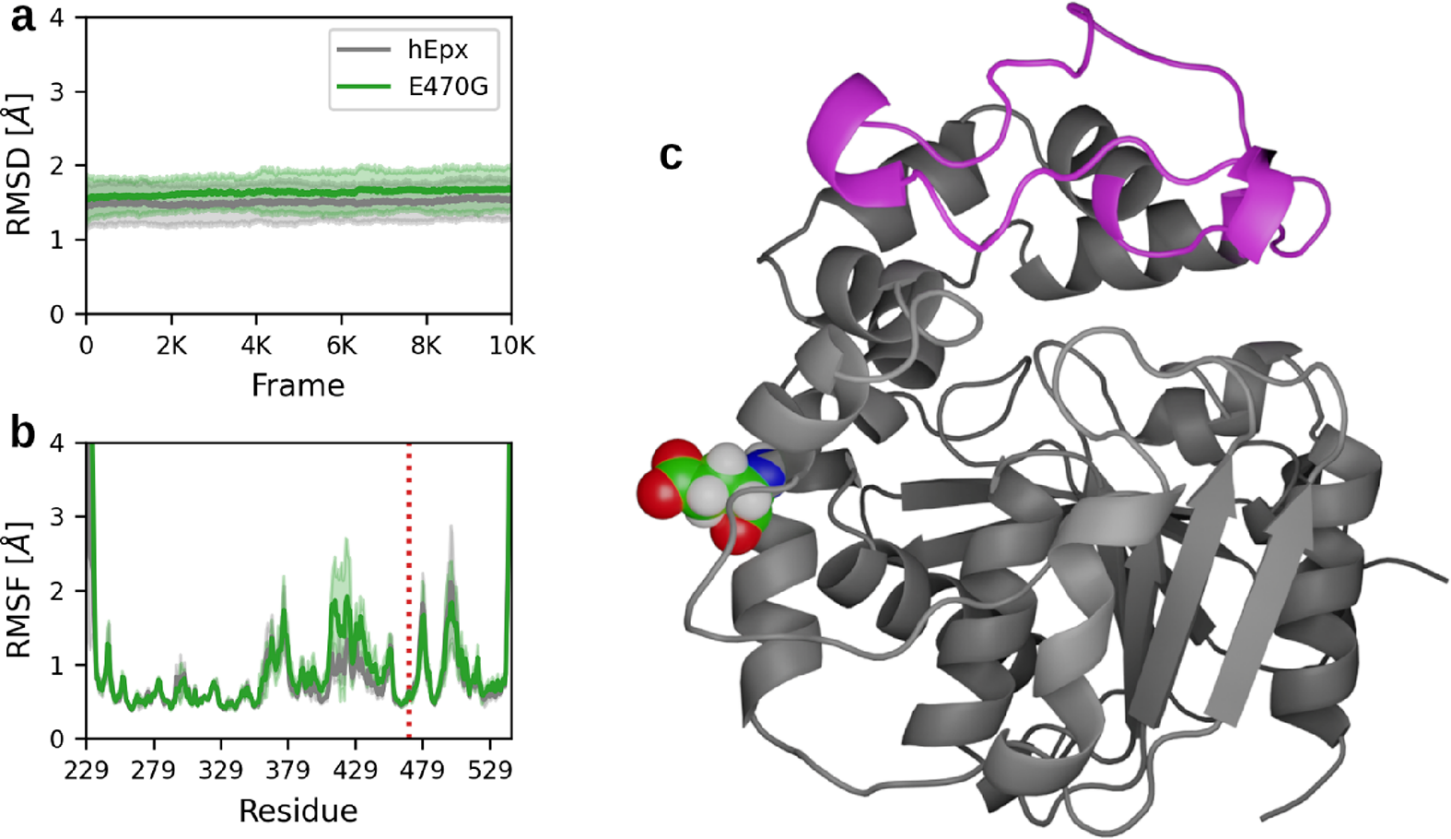
Stability and flexibility of the human epoxide hydrolase wild type and E470G mutant. (a) Average root-mean-square deviation and (b) root-mean-square fluctuation are depicted as solid lines, with standard deviation represented by shaded regions for 5 μs adaptive molecular dynamics simulations. The position of the mutation is indicated by a vertical dotted red line in (b). (c) Cartoon representation of hEpx with the most flexible region in magenta and the mutated residue represented as spheres.

Results from 5 μs MD simulations for each variant (hEpx and E470G) revealed minimal average differences in RMSD (Figure 6a) across all simulations (Figures S27 and S28). Similarly, the average RMSF values for both variants exhibited negligible differences, except for the region spanning S407 and E446 (Figure 6b), corresponding to a loop region on the cap domain of the protein (Figure 6c). This region demonstrated significantly increased fluctuations in E470G simulations compared with hEpx (Figures S29 and S30), indicating enhanced flexibility resulting from the mutation, despite the mutation not occurring within this region (Figure 6b,c). The distribution of *minimal event sphere* radii for hEpx and E470G shows a bell-shaped distribution, peaking at ∼1.6 Å in both cases (Figure S31). On the cumulative distribution of events, the proportion of events below 1.4 Å decreased to 12.4 and 13.8% in hEpx and E470G, respectively (Figure S31), compared with Hal, Epx, and Lip. This reduction in transport events may be attributed to a substantial increase in total transport events between proteins (30 times higher compared to Hal). The distribution of events by tunnels revealed that some tunnels preferred narrow radii, shifting the distribution away from the major tunnel (Figure S33), although the distribution was not as pronounced as observed for Epx or Hal (Figure S12). H-bonding analyses exhibited similar patterns and trends observed in Hal, Epx, and Lip, with a predominance of H-bonds at lower *minimal event sphere* radii (Figure S34) and the backbone atoms being the major contributors to such contacts (Figure S35).

The MD simulation results encompass all tunnels in hEpx documented in the literature,^65,71,72^ along with four newly identified ones (Table 1 and Figures S36–S38). When focusing solely on tunnels utilized for water transport, the *Tc/m* tunnel emerged as the major contributor in both variants (Figure 6a and Table S3), although its significance decreased by ∼16% in E470G compared to hEpx. Intriguingly, in a recent study of epoxide hydrolases,^72^ the *Tm1* tunnel was identified as the primary contributor to water transport in hEpx. However, this discrepancy may result from shorter simulation time and the water model employed (TIP3P versus OPC),^86,87^ which is in line with markedly different water transport observed for the Hal system (Figure S17b).

**Table 1:** Utilization of Tunnels Found in hEpx and E470G Mutant

**tunnel**	**tunnel IDs**	**water transport**		
		**WT**	**E470G**	**difference**
*Tm1*	2, 4, 8	22.37%	24.48%	–2.12%
*Tm2*	16	0.83%	2.45%	–1.62%
*Tm3*	3, 20, 37	4.66%	5.90%	–1.25%
*Tm5■*	6	5.14%	1.66%	**3.49%**
*Tc/m*	1, 29, 34	55.86%	39.63%	16.22%
*Tg*	12	0.76%	1.40%	–0.64%
*Tside●*	5, 7	9.13%	17.49%	**-8.37%**
*Tcap1⊥*	14, 24	0.00%	2.27%	**-2.27%**
*Tcap2*	15	0.01%	0.10%	–0.08%
*Tcap4*	9, 39	0.09%	0.04%	0.05%
*Tnew_A★*	10	0.21%	4.20%	**-3.98%**
*Tnew_B*	11	0.03%	0.02%	0.01%
*Tnew_C*	22	0.28%	0.16%	0.12%
*Tnew_D*	23	0.62%	0.19%	0.43%

**Figure 7:**
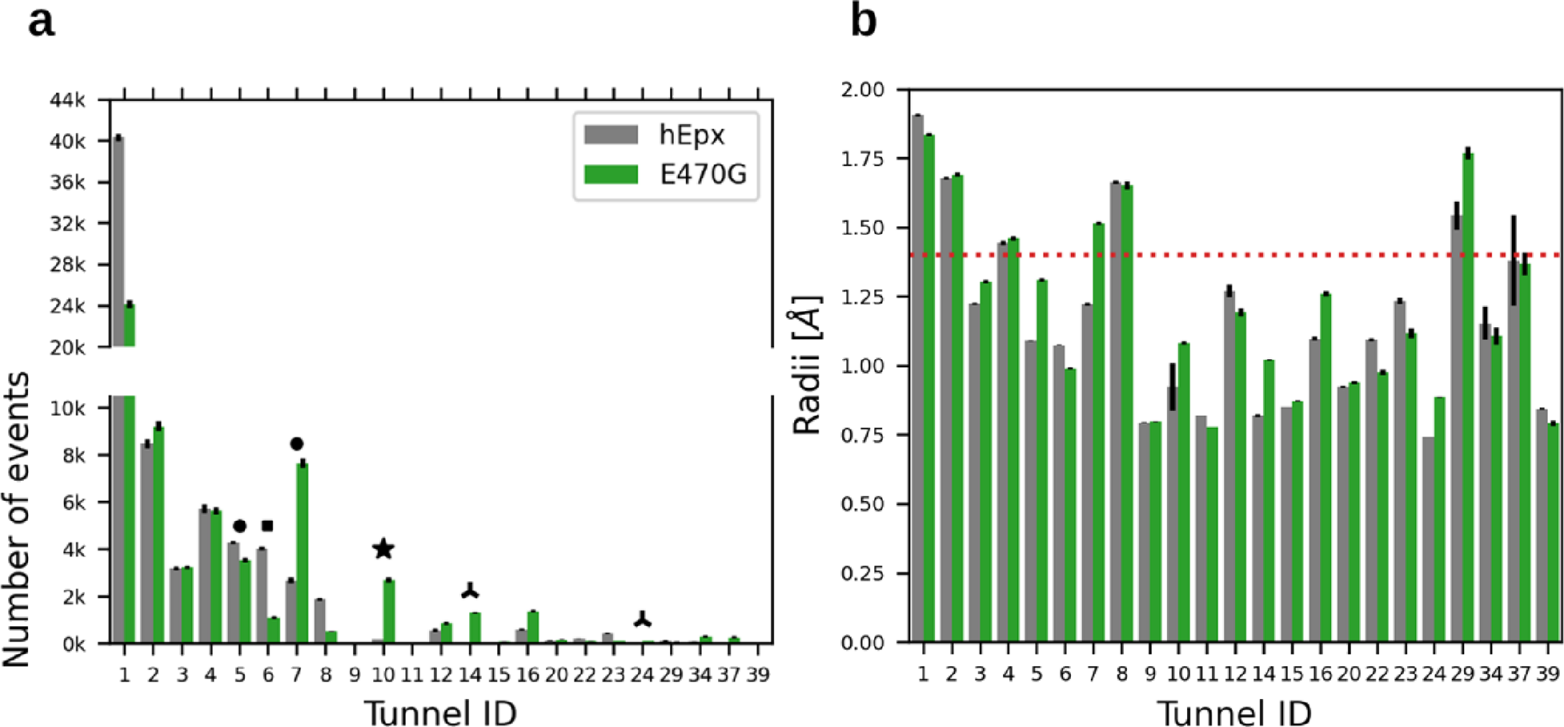
Tunnels employed by water in the human epoxide hydrolase wild type and E470G mutant. (a) Total number of transport events by the tunnel for hEpx and E470G. (b) Average bottleneck radius for tunnels employed by water in hEpx and E470G, with the accepted radius for a water molecule marked by a horizontal red dotted line. Narrow tunnels with altered water transport are denoted by black shapes corresponding to the names in Table 1. The reported variances were estimated using jackknife resampling from 50 contributing simulations.^88^

An examination of the average bottleneck radius of the tunnels reveals that most have a radius below the widely accepted 1.4 Å radius for a water molecule (Figure 7b and Table S3). Although it is expected that the *Tm1* and *Tc/m* tunnels, with larger bottleneck radii, transport approximately 78 and 64% of total water in hEpx and E470G, respectively (Tables 1 and S3), the contribution of narrower tunnels should not be dismissed, particularly in E470G, where the mutation appears to have significantly altered tunnel usage (Table 1 and Table S3). In nearly all tunnels, the volume of transported waters increased in E470G compared to hEpx (Table S3), likely due to reduced transport via the *Tc/m* tunnel (Figure 7a and Table 1). A noteworthy case resulting from the mutation is the newly identified *Tnew_A* tunnel, whose bottleneck radius increased by 0.128 Å, while its importance for water transport increased 20-fold (Table 1 and Table S3). The mutation also opened the *Tcap1* tunnel and increased the flow in the *Tside* tunnel in E470G (Figure 7a and Table 1). Similarly, a minimal reduction in the bottleneck radius of the *Tm5* tunnel by 0.085 Å in the E470G mutant led to a nearly 25% decrease in transport compared to the wild-type hEpx (Figure 7a and Table 1).

We hypothesize that the modified water transport and tunnel usage by hEpx and E470G may contribute to the phenotypic changes observed in these variants. The reduced water flow in E470G could create a more suitable environment for catalysis by reducing the competition of water molecules as nucleophiles in the second chemical step or enhancing the rate of the first chemical step leading to the formation of alkylenzyme intermediates, which often determines the overall catalytic efficiency for epoxide hydrolases,^89,90^ potentially leading to the increased activity observed in experimental tests.^39^ Although a comprehensive structural explanation for the observed differences in these two variants is beyond the scope of this paper, we propose that the discrepancies in water flow could offer insights into understanding their phenotypes. Moreover, the entry of a few water molecules into the active site can significantly enhance the overall efficiency of LinB86 dehalogenase,^91^ where the new inflow of water molecules through the engineered narrow tunnel notably facilitates the rate-limiting product release. It is important to clarify that differences in water transport between hEpx and E470G cannot be considered a direct cause of an increased risk of an ischemic stroke. However, these differences provide more nuanced insights than a simple conformational analysis, demonstrating increased flexibility in certain regions. Furthermore, variations in tunnel usage were identified in tunnels with bottleneck radii that might be easily overlooked if the focus is solely on wider tunnels. These findings also expand potential targets for protein engineering, where the opening or closing of tunnels has been successfully employed to alter relevant functional properties of enzymes.^91^

## Conclusions

In this paper, we have demonstrated that water molecules can traverse extremely narrow tunnels, even with subangstrom radii. Approximately 20% of the events in the evaluated proteins involved such narrow tunnels, reconciling the discrepancy between geometry-based and ligand-tracking methods for tunnel identification.^27^ We also illustrated that to overcome repulsion during transport, water molecules establish multiple H-bonds, primarily with the backbone. Furthermore, we observed that the number of H-bonds formed is inversely proportional to the *minimal event sphere* radius. Although we currently lack sufficient data to support the extrapolation of these observations beyond the α/β-hydrolase fold, our findings suggest a need to reconsider classical limits on the functional tunnels in enzymes, moving beyond the pure dimensions of their bottleneck. Lastly, based on the insights gained, we propose a structural and functional hypothesis for the observed differences in hEpx and a single-point mutant variant. These differences can be traced to minor alterations in tunnel dimensions, resulting in a major rewiring of water transport flows through the active site.

## Data Availability

The underlying data for this study are available in the published article, the Supporting Information, and in Zenodo repositories at 10.5281/zenodo.7966081 (Hal), 10.5281/zenodo.7966058 (Epx), 10.5281/zenodo.7966091 (Lip), 10.5281/zenodo.7965660 (hEpx), 10.5281/zenodo.7965880 (E470G), 10.5281/zenodo.10911961 (Hal with different MD simulation settings), and 10.5281/zenodo.10911789 (water–protein interaction analyses). The data include Python3 scripts, binary files, plain text, and PDB-formatted and AMBER-formatted structural data, all compatible with various freely available SW packages. No tools with restricted access are required.

## Supporting Information Available

RMSD and RMSF plots for adaptive simulations of Hal (under four different simulation setup), Epx, Lip, hEpx, and E470G; visual representation of tunnels identified in adaptive simulations of Hal, Epx, Lip, hEpx, and E470G; distribution of *minimal event sphere* radii in Hal, Epx, Lip, hEpx, and E470G concerning tunnels, H-bonds, and interacting atoms; comparison of distributions of *minimal event sphere* radii in Hal under four different simulation setups; closest surface–surface distances between protein atoms and water molecules present in individual bins of *minimal event sphere* radii in Hal, Epx, and Lip; binding free energy of water molecules present in individual bins of *minimal event sphere* radii in Hal, Epx, and Lip calculated by MM/GBSA; distribution of *minimal event sphere* radii in hEpx and E470G; literature-based naming of tunnels identified in Hal, Epx, and Lip; number of events detected and assigned to tunnels in Hal, Epx, and Lip; and water usage and average bottlenecks of tunnels in hEpx and E470G (PDF)

## Supplementary Material

ci4c00094_si_001.pdf

ci4c00094_si_002.avi

## Abbreviations

Hal: haloalkane dehalogenase from *Rhodococcus rhodochrous*
Epx: potato epoxide hydrolase 1
Lip: lipase from *Diutina rugosa* (previously *Candida rugosa*)
MD: molecular dynamics
hEpx: human epoxide hydrolase
E470G: E470G mutant of human epoxide hydrolase
MSM: Markov state models
RMSD: root-mean-square deviation
RMSF: root-mean-square fluctuation
3D-RISM: 3D reference interaction site model
PDB: Protein Data Bank
HTMD: High-throughput molecular dynamics
H-bonds: hydrogen bonds
HMR: hydrogen mass repartitioning
MM/GBSA: molecular mechanics/generalized Born surface area
