# Supplementary material for: Water will Find Its Way: Transport through Narrow Tunnels in Hydrolases
Source: J Chem Inf Model. 2024 Apr 26;64(15):6014–25. doi: 10.1021/acs.jcim.4c00094 (PMC11323245; doi:10.1021/acs.jcim.4c00094)
Supplement: Supplementary file 1 — ci4c00094_si_001.pdf [file ci4c00094_si_001.pdf]

SUPPORTING INFORMATION:

Water will find its way: transport through narrow tunnels in hydrolases

Carlos Sequeiros-Borja[1,2], Bartlomiej Surpeta[1,2], Aravind Selvaram Thirunavukarasu[1,2], Cedrix J. Dongmo Foumthuim[3], Igor Marchlewski[1,2], Jan Brezovsky[1,2]*

1 International Institute of Molecular and Cell Biology, Warsaw, Poland

2 Laboratory of Biomolecular Interactions and Transport, Department of Gene Expression, Institute of Molecular Biology and Biotechnology, Faculty of Biology, Adam Mickiewicz University, Poznań, Poland

3 National Institute of Nuclear Physics (INFN), Sezione di Roma Tor Vergata, Rome, Italy

Corresponding author: Jan Brezovsky, E-mail: janbre@amu.edu.pl, jbrezovsky@iimcb.gov.pl

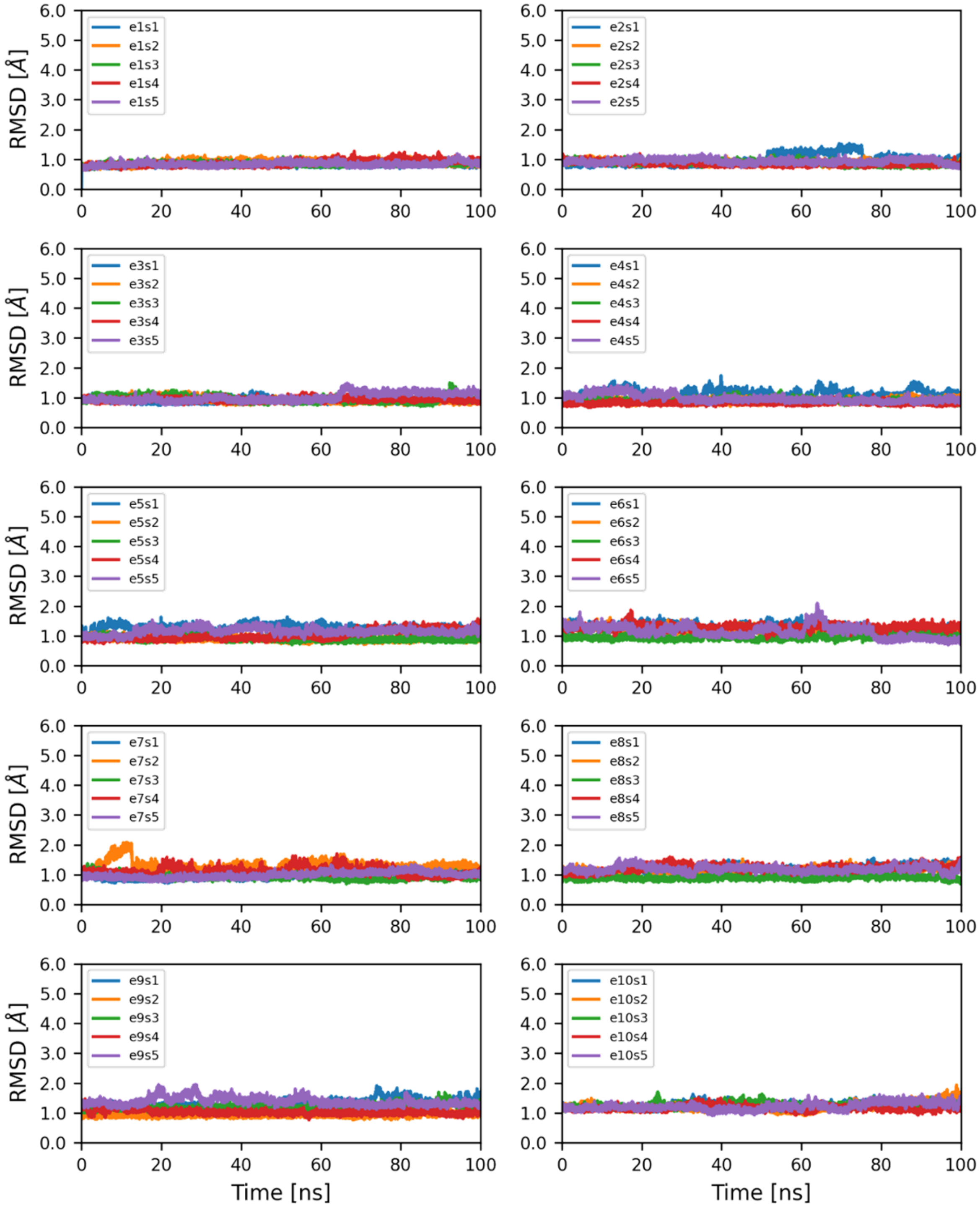

Figure S1. RMSD of Hal in adaptive MD simulations. 50 MD simulations of Hal grouped by epochs.

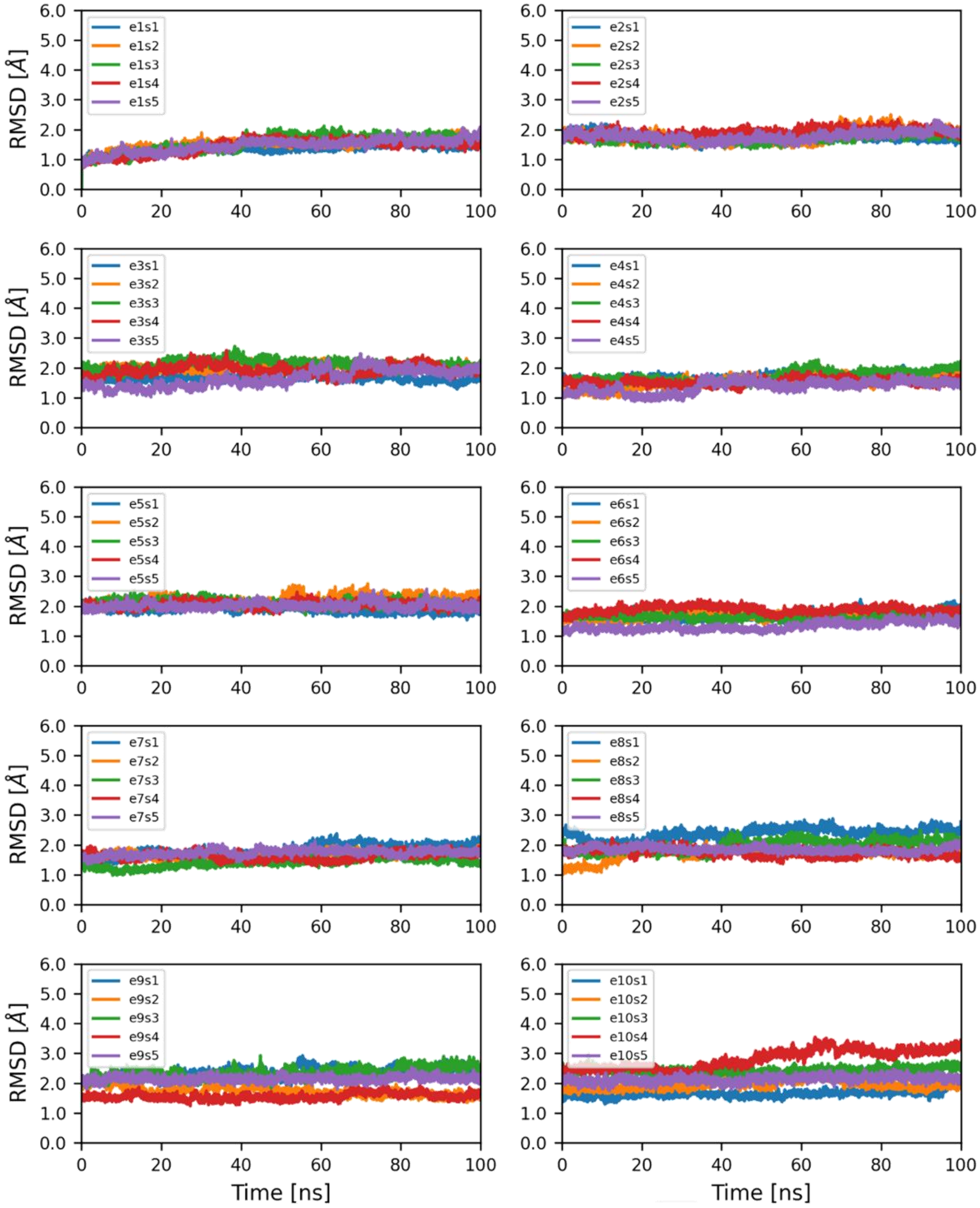

Figure S2. RMSD of Epx in adaptive MD simulations. 50 MD simulations of Epx grouped by epochs.

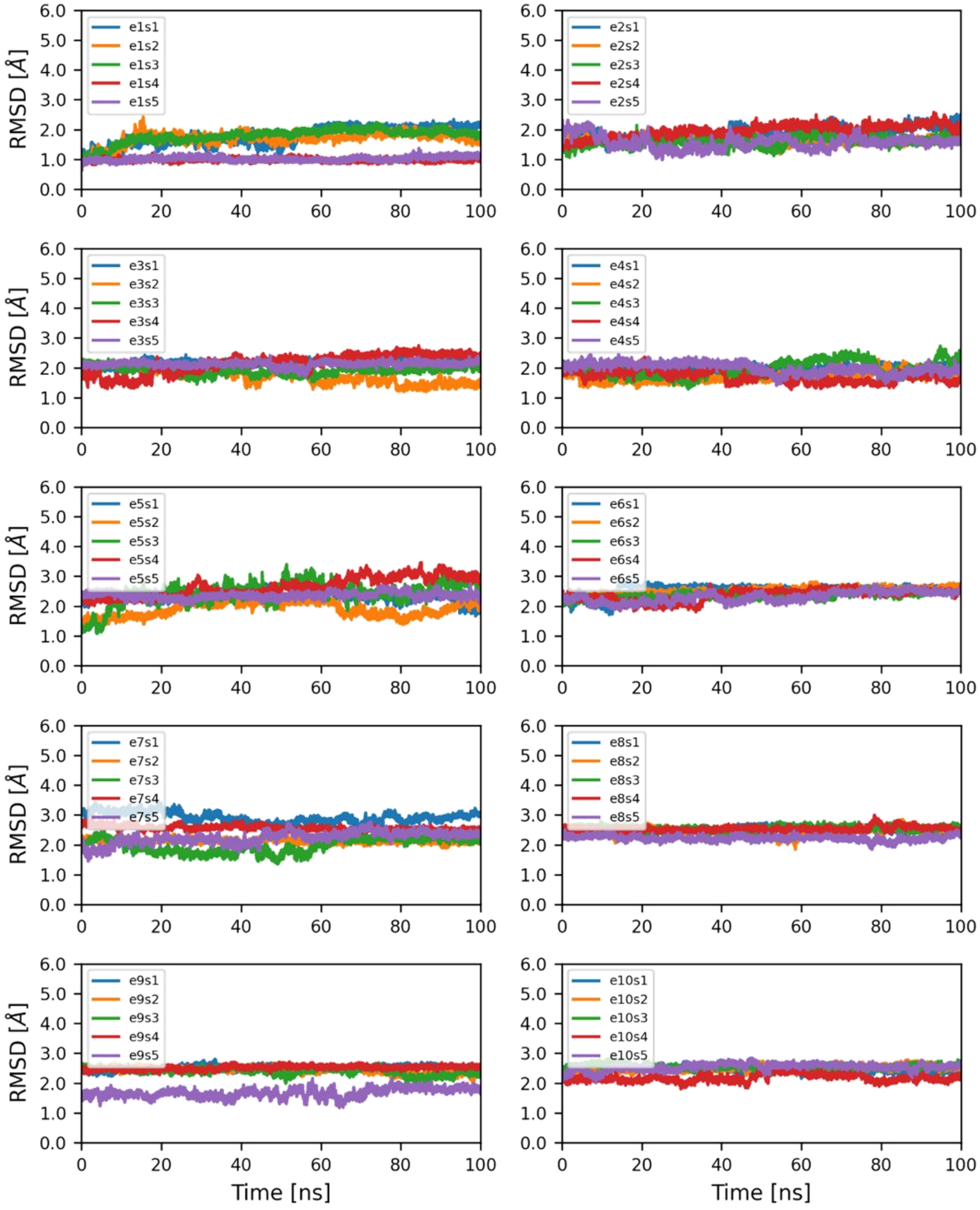

Figure S3. RMSD of Lip in adaptive MD simulations. 50 MD simulations of Lip grouped by epochs.

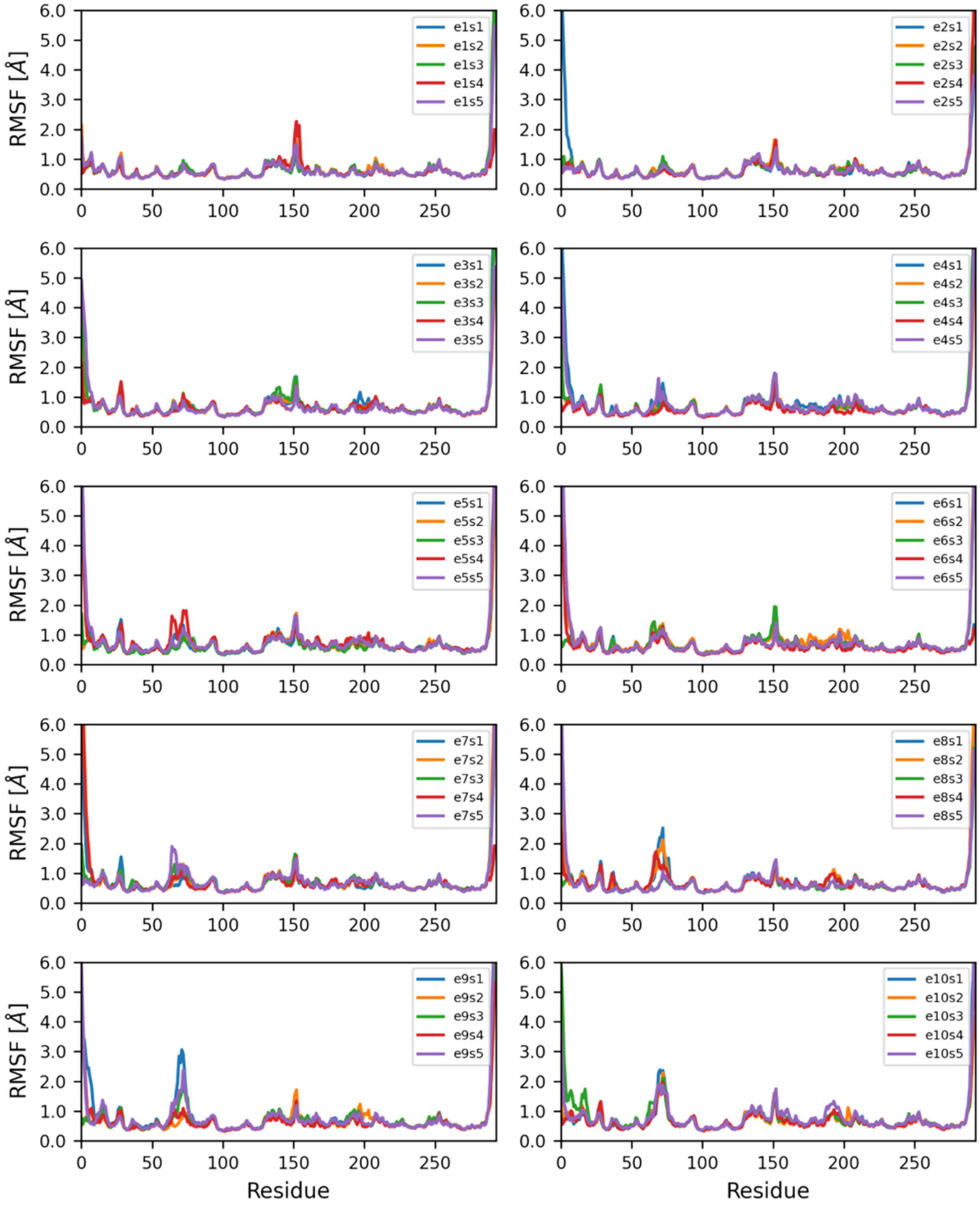

Figure S4. RMSF of Hal in adaptive MD simulations. 50 MD simulations of Hal grouped by epochs.

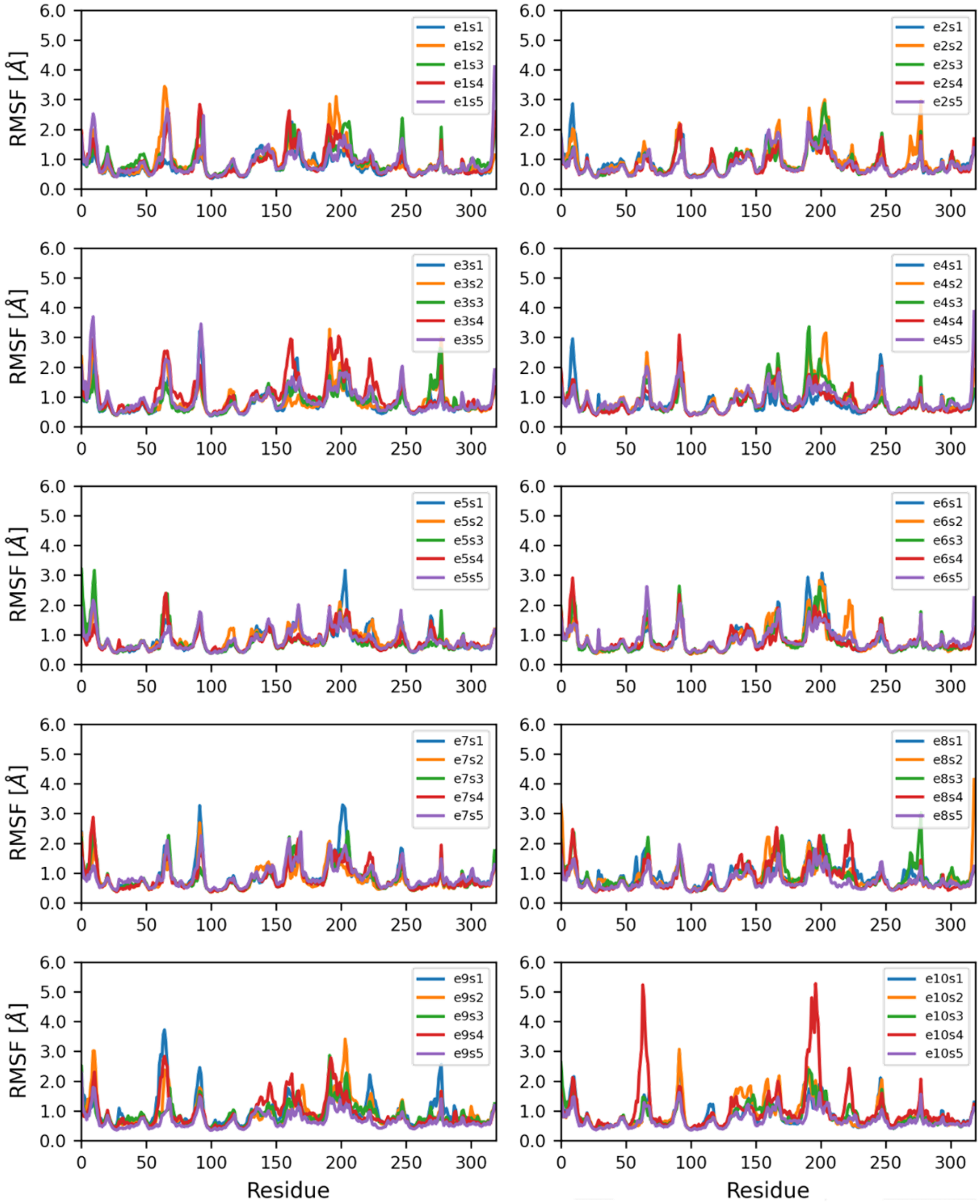

Figure S5. RMSF of Epx in adaptive MD simulations. 50 MD simulations of Epx grouped by epochs.

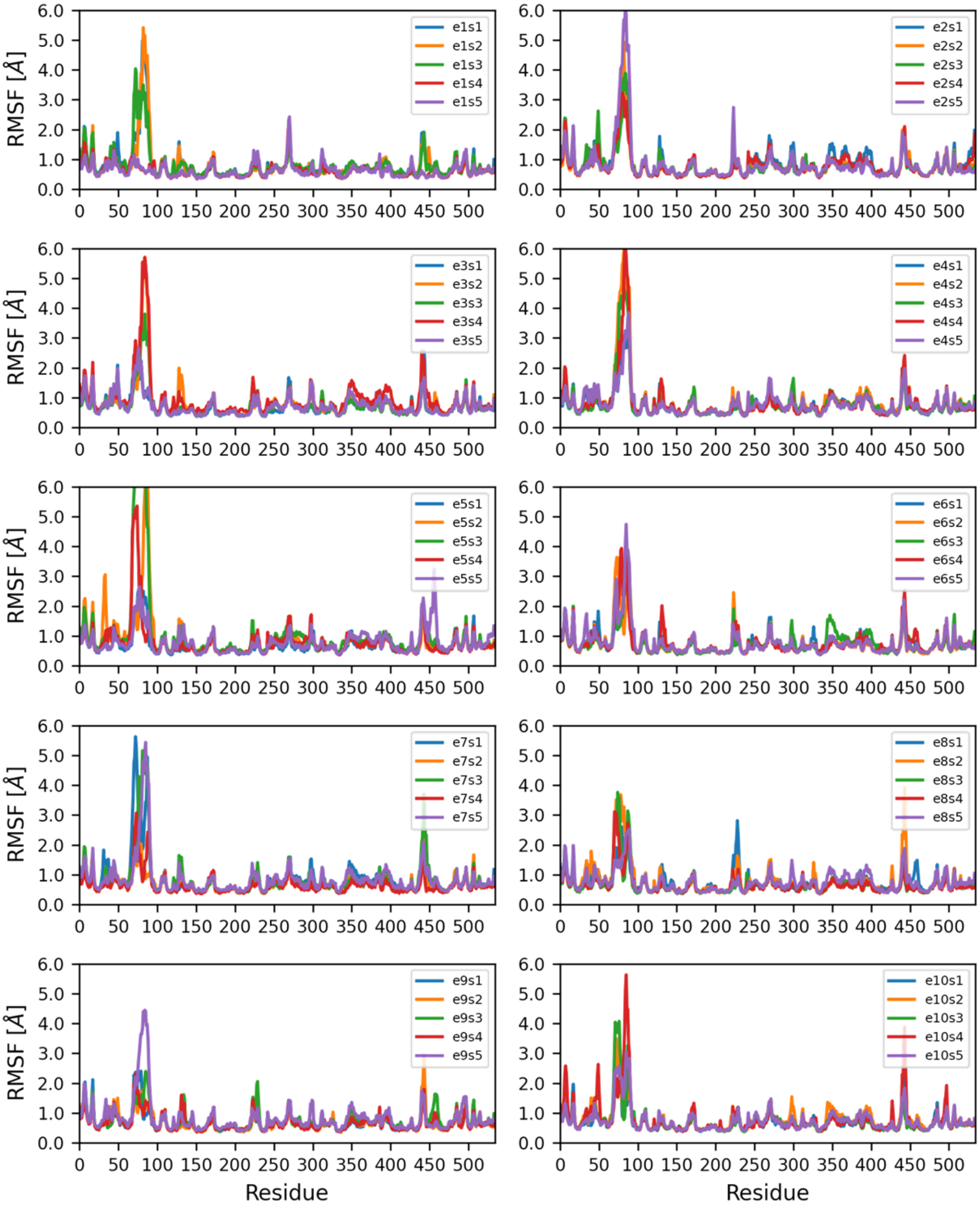

Figure S6. RMSF of Lip in adaptive MD simulations. 50 MD simulations of Lip grouped by epochs.

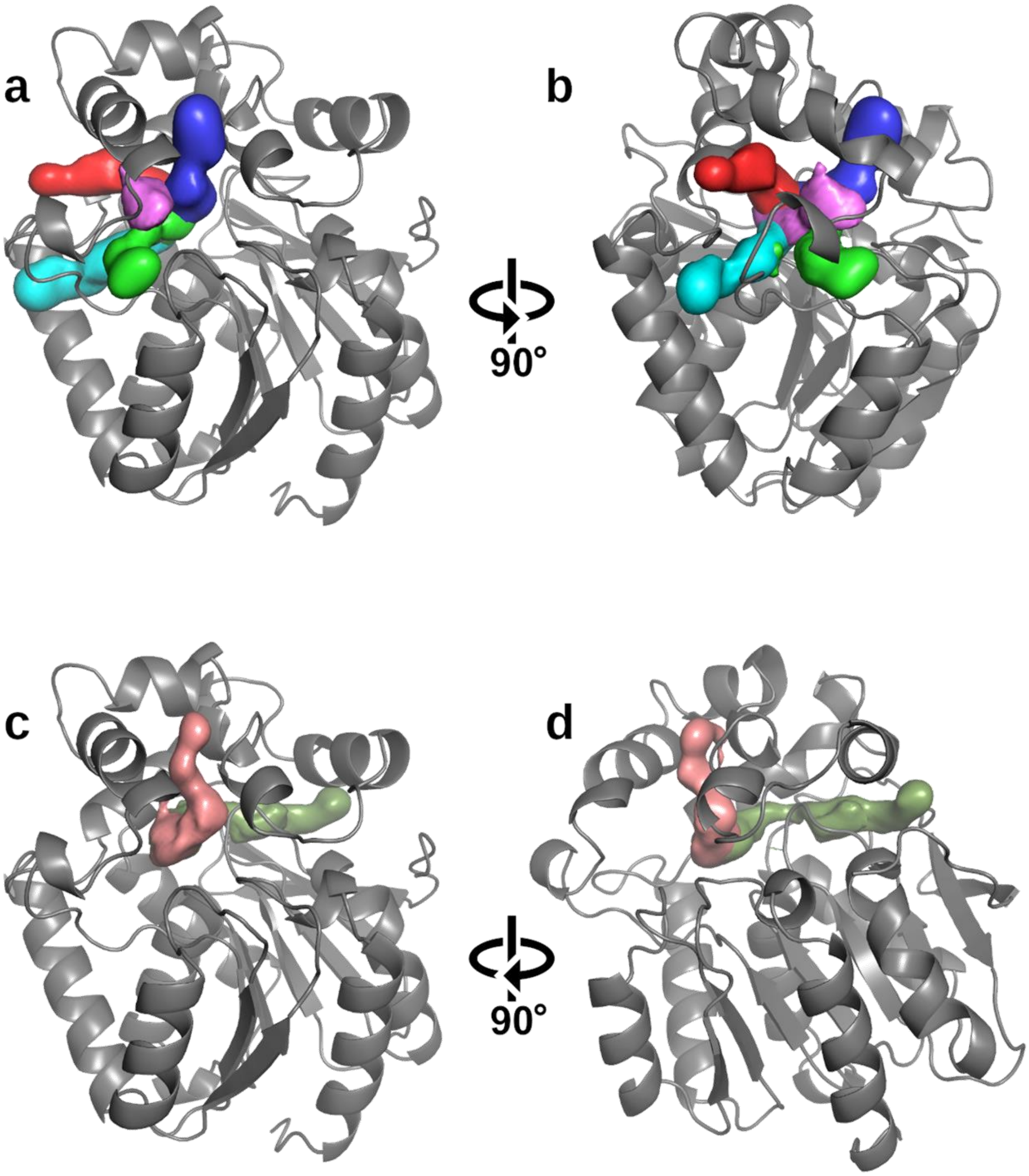

Figure S7. Tunnels identified in Hal. Tunnels' densities at 20% of presence in Hal obtained from 5 µs adaptive MD simulations. Tunnels described in the literature: a) *p1* (blue), *p2a* (green), *p2b* (pink), *p2c* (cyan), and *p3* (red); b) rotating the same view 90° counterclockwise in the Z-axis; and newly observed tunnels: c) *up* (salmon) and *side* (light green) tunnels; d) rotating the same view 90° clockwise in the Z-axis.

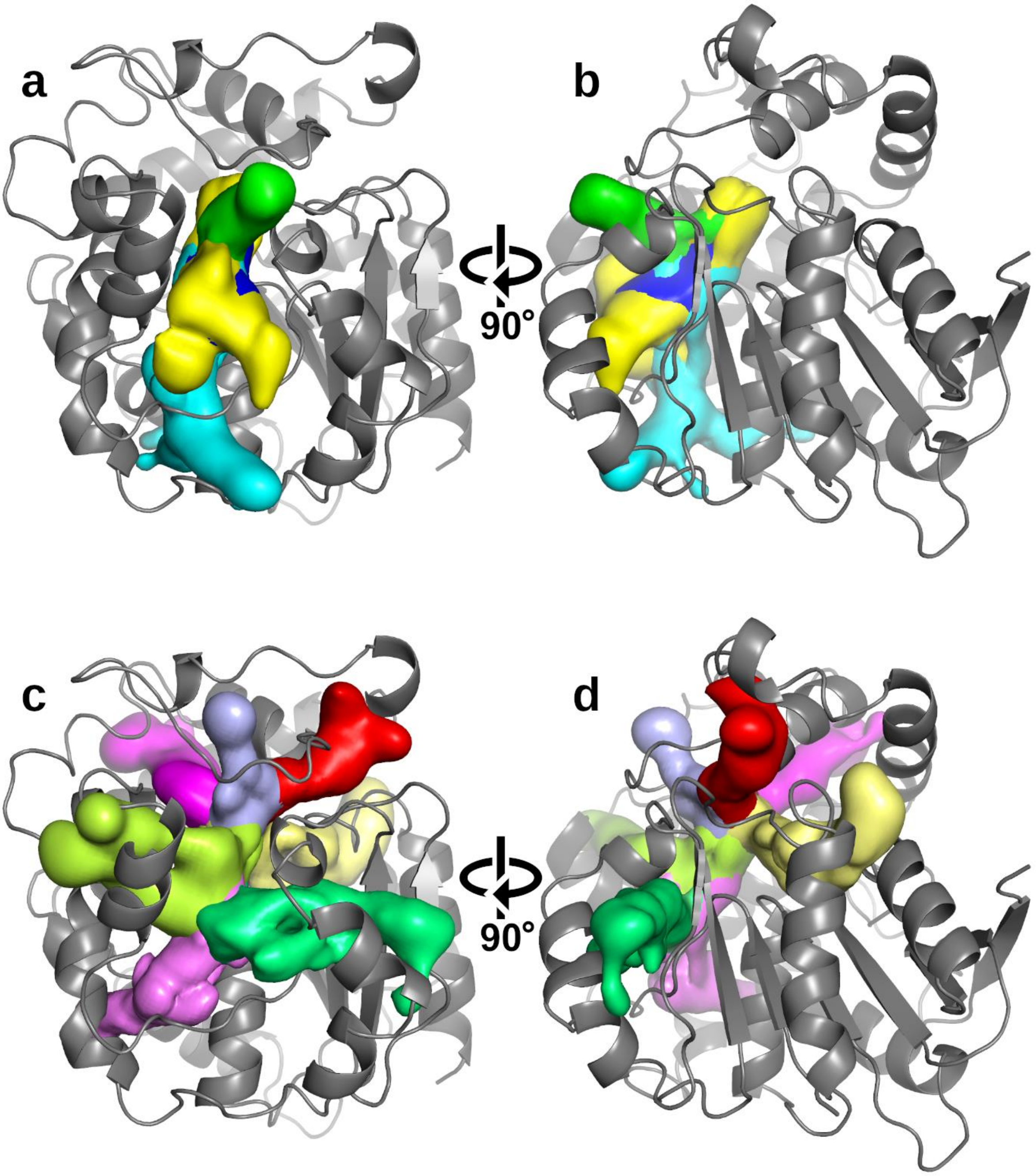

Figure S8. T**unnels identified in Epx.** Tunnels' densities at 20% of presence in Epx obtained from 5 µs adaptive MD simulations. Tunnels described in the literature: a) *TM1* (blue and yellow), *TC/M* (green), and *TM2* (cyan); b) rotating the same view 90° clockwise in the Z-axis. Newly identified lesser tunnels that also participate in water transport: c) and the same view d) after rotating the view 90° clockwise in the Z-axis (4, 6, 8, 14, 15, 16, and 21 in red, magenta, light-blue, lemon, pale-yellow, violet, and lime respectively; IDs from **Table S1**).

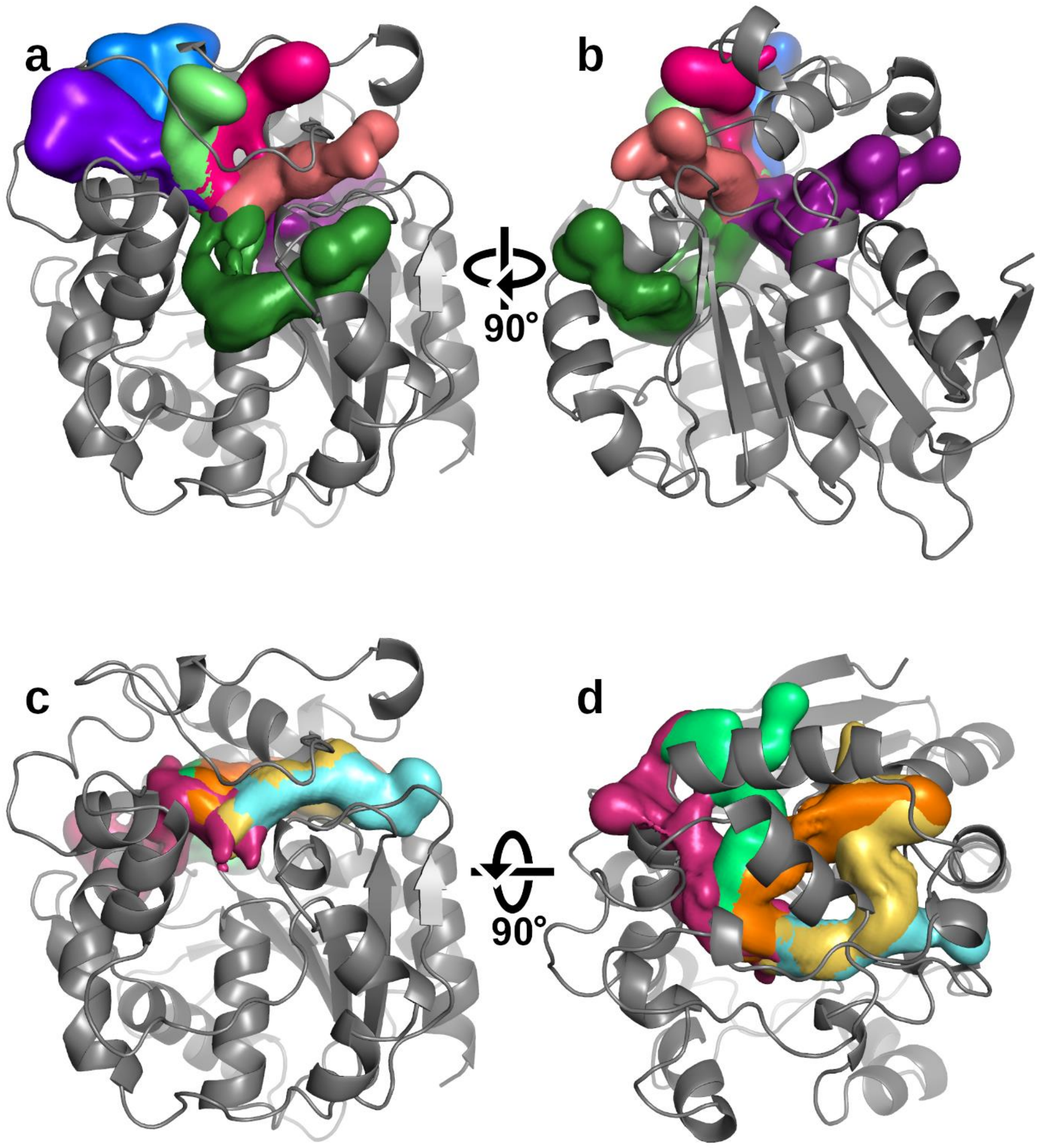

Figure S9. New water-conducting tunnels identified in Epx. Tunnels' densities at 20% of presence in Epx obtained from 5 µs adaptive MD simulations. a) and same view b) after rotating the view 90° clockwise in the Z-axis (9, 10, 11, 12, 13, 22, and 25 in blue, light-green, salmon, fuchsia, purple, dark-green, and wine, respectively; IDs from **Table S1**). More tunnels identified c) and the same view d) after rotating the view 90° on the X-axis (7, 17, 19, 23, and 24 in lime, orange, aquamarine, dark-pink, and pale-yellow, respectively; IDs from **Table S1**).

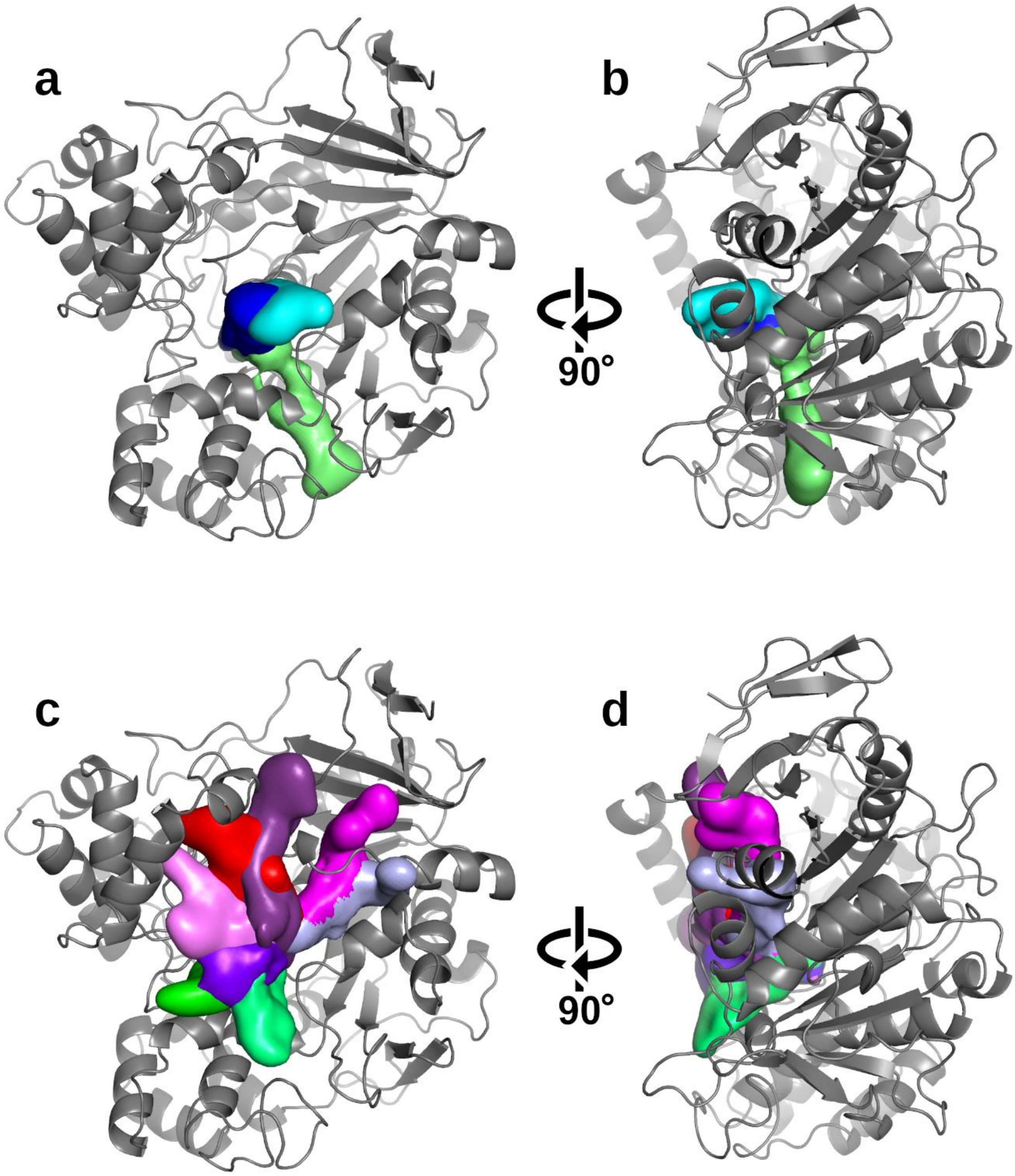

Figure S10. Tunnels identified in Lip. Tunnels' densities at 20% of presence in Lip obtained from 5 µs adaptive MD simulations. Tunnels described in the literature: a) the *main* tunnel (blue and cyan) and *ester* tunnel (pale green); b) rotating the same view 90° clockwise in the Z-axis. Newly identified lesser tunnels that also participate in water transport: c) and the same view d) after rotating the view 90° clockwise in the Z-axis (2, 3, 5, 8, 11, 12, 14, and 16 in red, green, magenta, light-blue, lime, purple, dark-purple, and pink, respectively; IDs from **Table S1**).

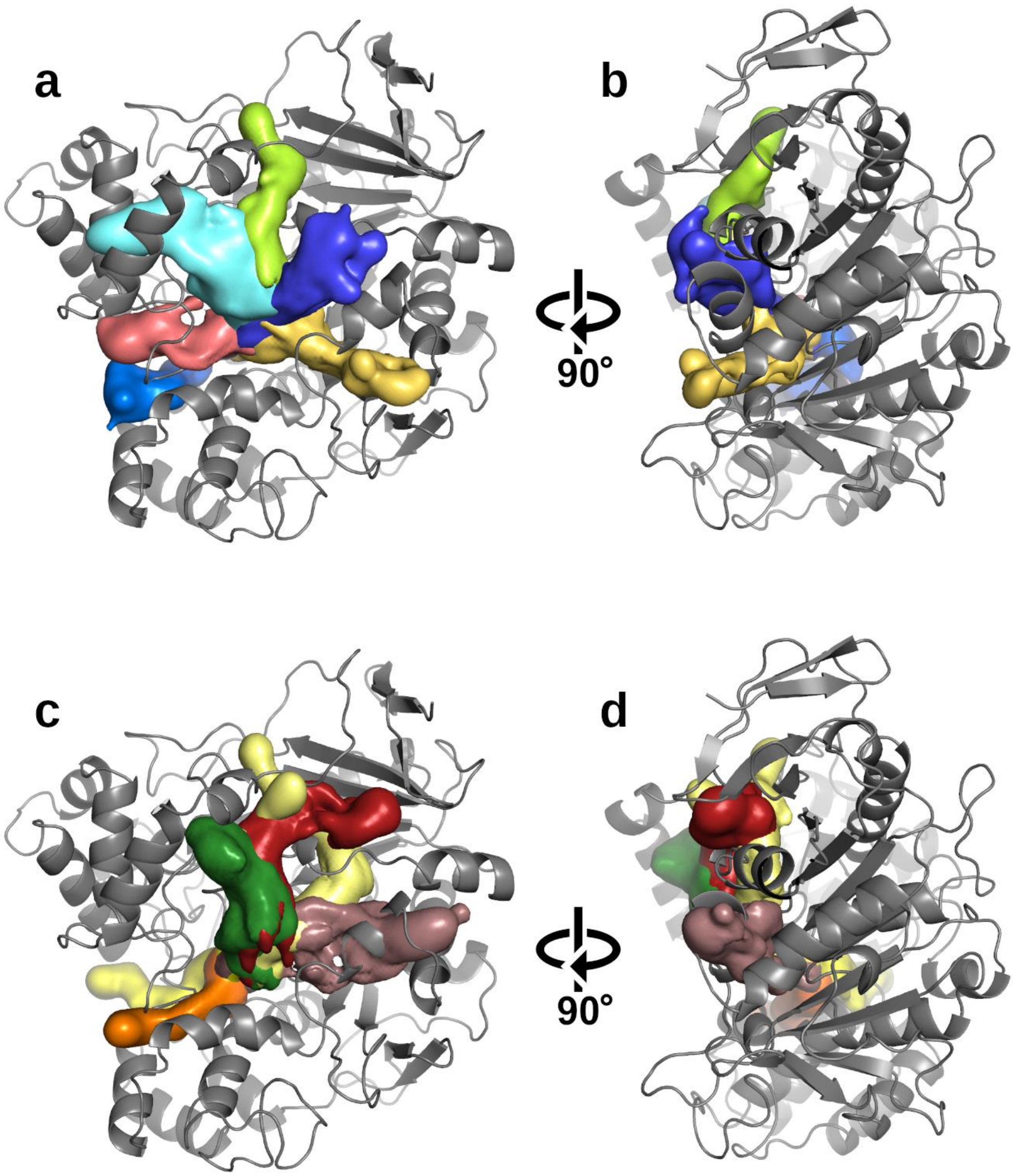

Figure S11. New water-conducting tunnels identified in Lip. Tunnels' densities at 20% of presence in Lip obtained from 5 µs adaptive MD simulations. a) and same view b) after rotating the view 90° clockwise in the Z-axis (9, 10, 15, 18, 24, and 26 in salmon, blue, lemon, cyan, pale-yellow, and dark-blue, respectively; IDs from **Table S1**). More tunnels identified c) and the same view d) after rotating the view 90° on the X-axis (6, 17, 19, 38, 66, and 70 in yellow, pale-yellow, orange, dark-red, dark-green, and pale-violet, respectively; IDs from **Table S1**).

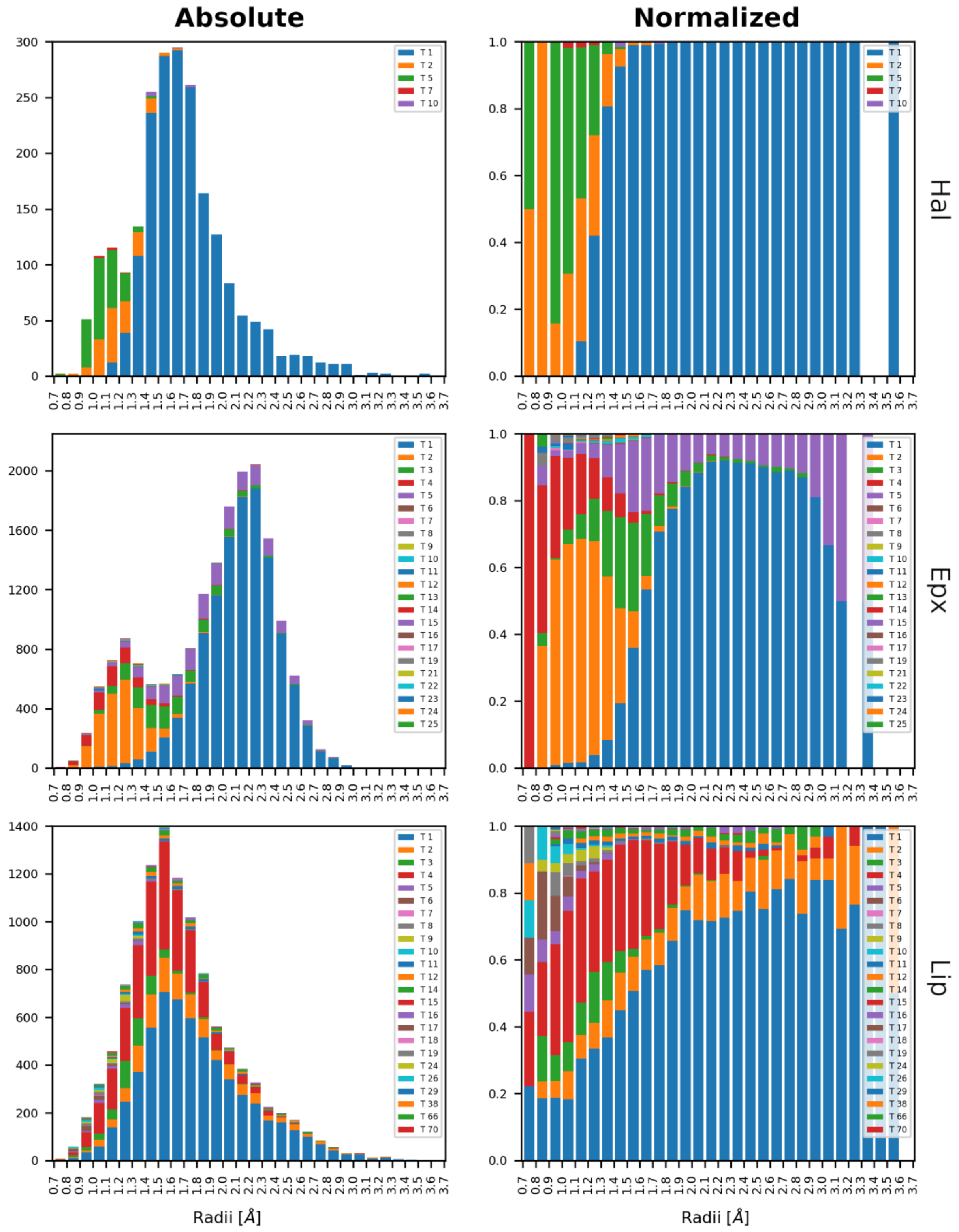

Figure S12. Distribution of *minimal event sphere* radii in Hal, Epx, and Lip by tunnels. Histograms of the radius at which a water event occurs are presented in absolute and normalized scales for Hal, Epx, and Lip. Colors represent individual clusters. However, it loops in Epx and Lip cases due to the high number of tunnels identified, IDs from **Table S1**.

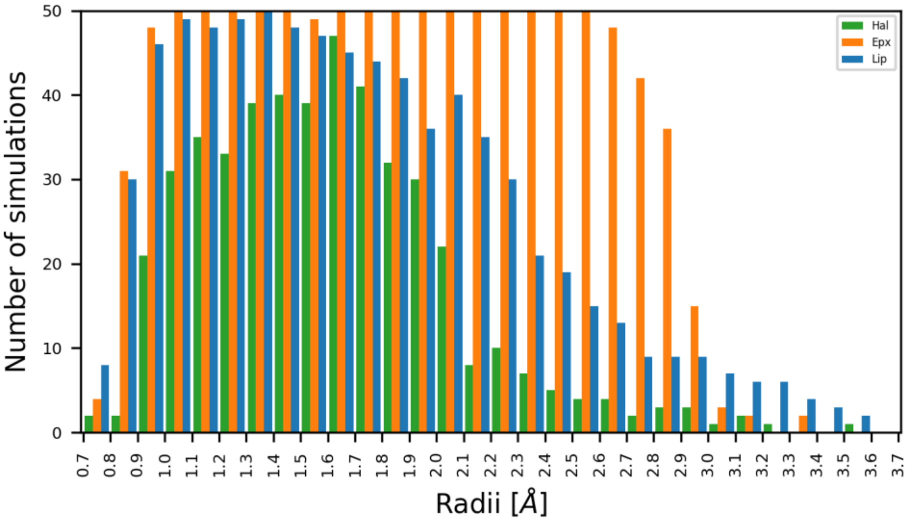

Figure S13. Number of MD simulations contributing events to each histogram bin in Hal, Epx, and Lip.

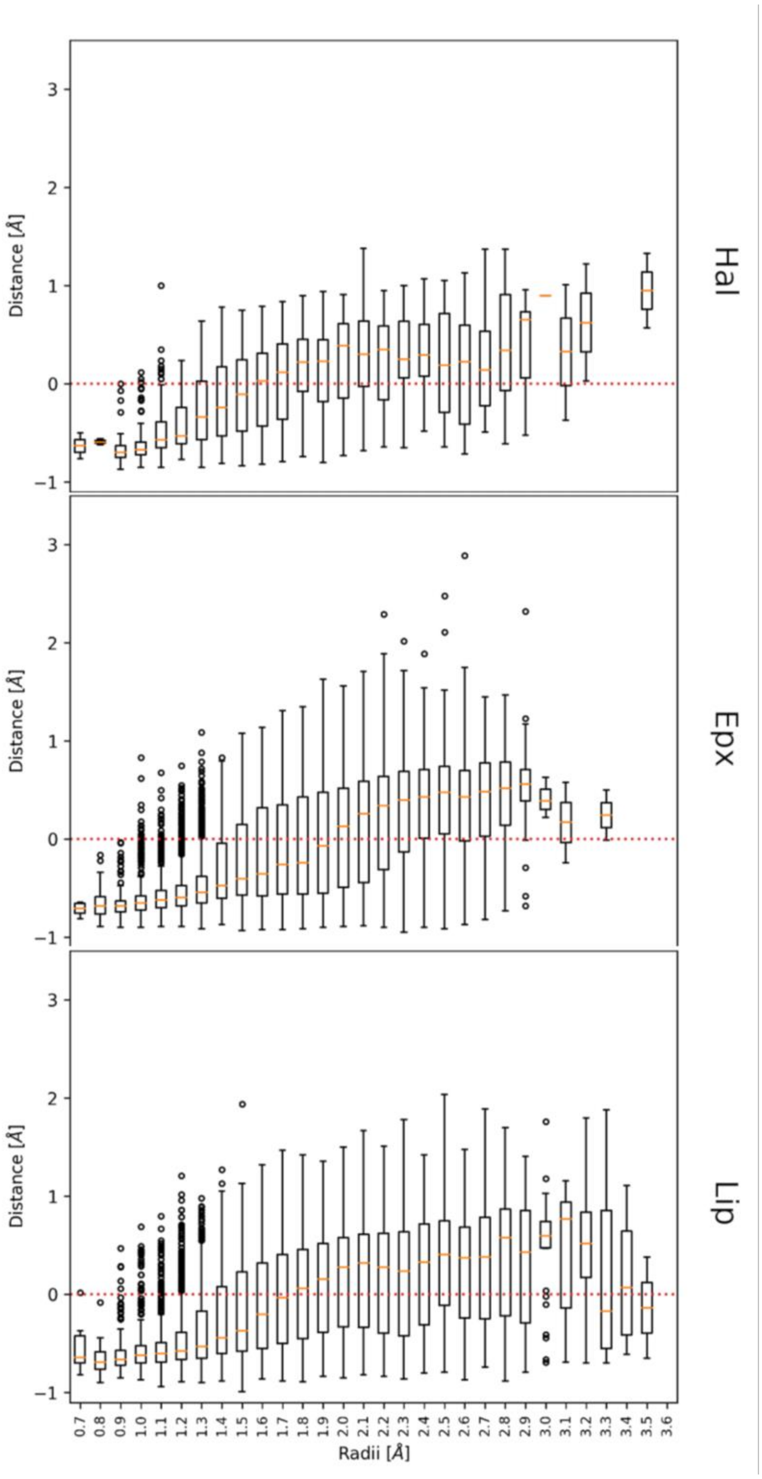

Figure S14. The surface-surface distances between closest protein atoms and water molecules present in individual bins of *minimal event sphere* radii in Hal, Epx, and Lip. These distances are calculated as distance between atom centers minus a sum of atomic radii. Hence, distances below the 0 Å threshold (red dotted line) represent the overlap between the closest protein and water atoms.

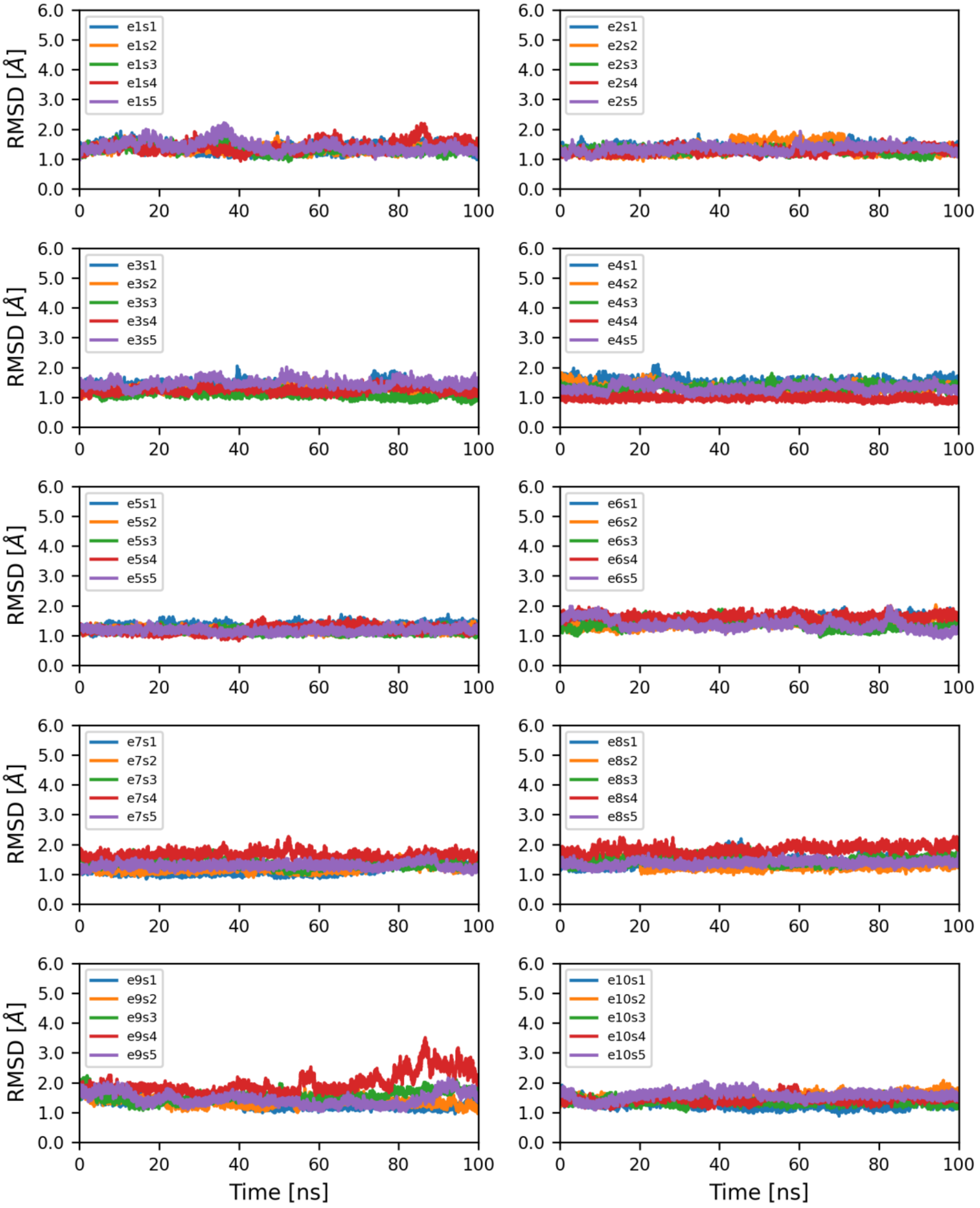

Figure S15. RMSD of Hal with 2-fs timestep and without HMR in adaptive MD simulations. 50 MD simulations of Hal with 2-fs timestep and without HMR grouped by epochs.

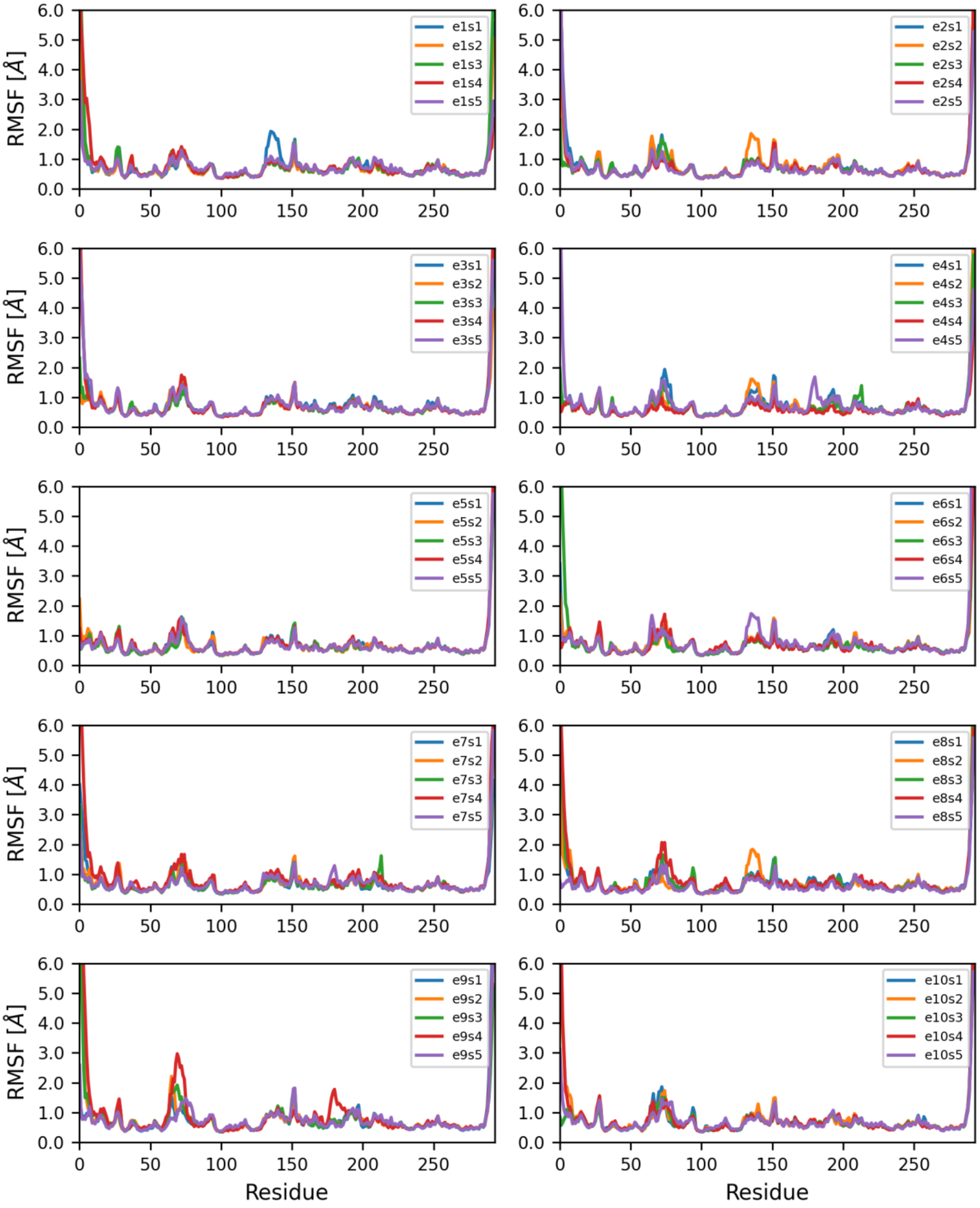

Figure S16. RMSF of Hal with 2-fs timestep and without HMR in adaptive MD simulations. 50 MD simulations of Hal with 2-fs timestep and without HMR grouped by epochs.

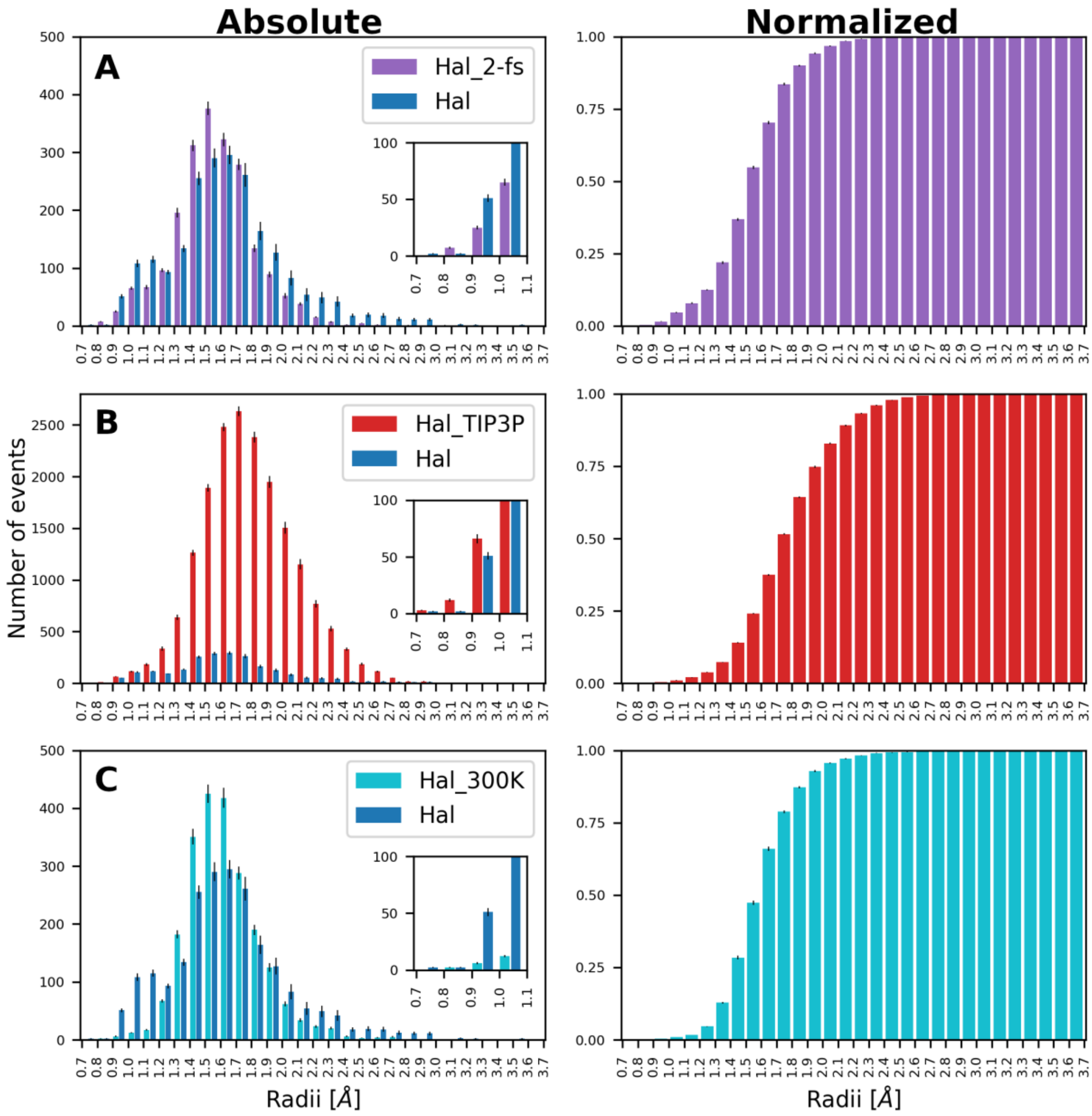

Figure S17. Distribution of *minimal event sphere* radii in Hal simulations under different setup. The absolute number of transport events and their corresponding normalized cumulative distribution is shown for different simulation setup: (**a**) simulations with 2-fs timestep and without HMR applied (Hal_2-fs), (**b**) simulations using TIP3P model for water molecules (Hal_TIP3P), and (**c**) simulations at lower temperature of 300 K (Hal_300K). The variances were estimated by resampling 100 times 45 out of 50 available MD simulations; the number of simulations contributing to each bin is shown in **Figure S18**.

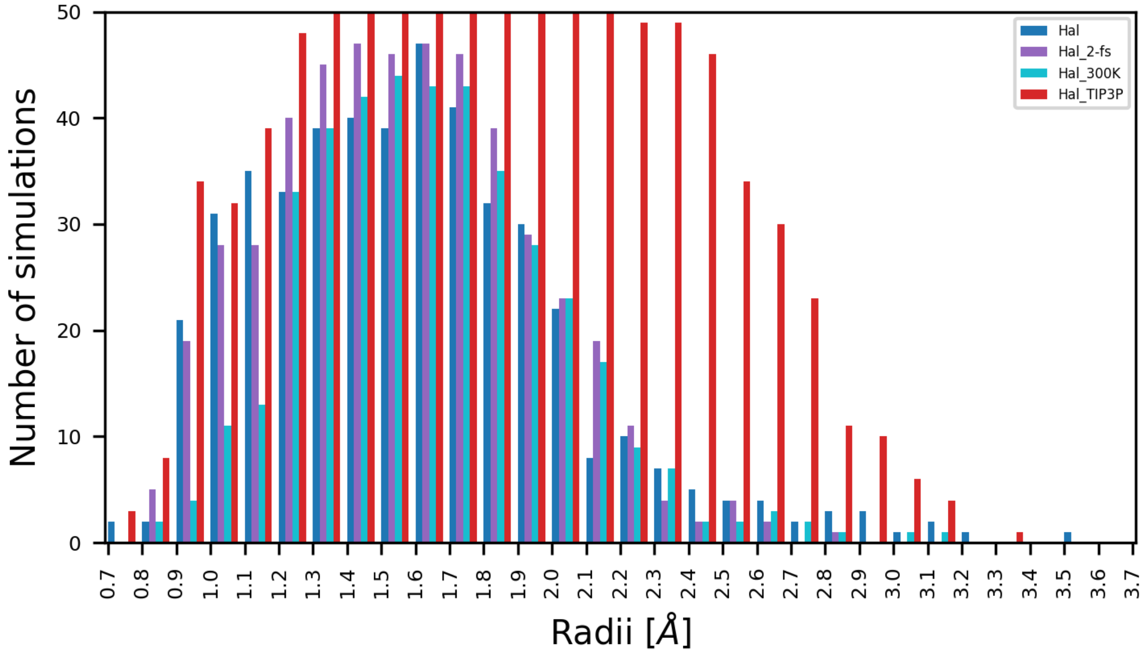

Figure S18. Number of MD simulations contributing events to each histogram bin in Hal simulations under different setup. This comprises of i) simulations with 2-fs timestep and without HMR applied (Hal_2-fs), ii) simulations at lower temperature of 300 K (Hal_300K), and iii) simulations using TIP3P model for water molecules (Hal_TIP3P).

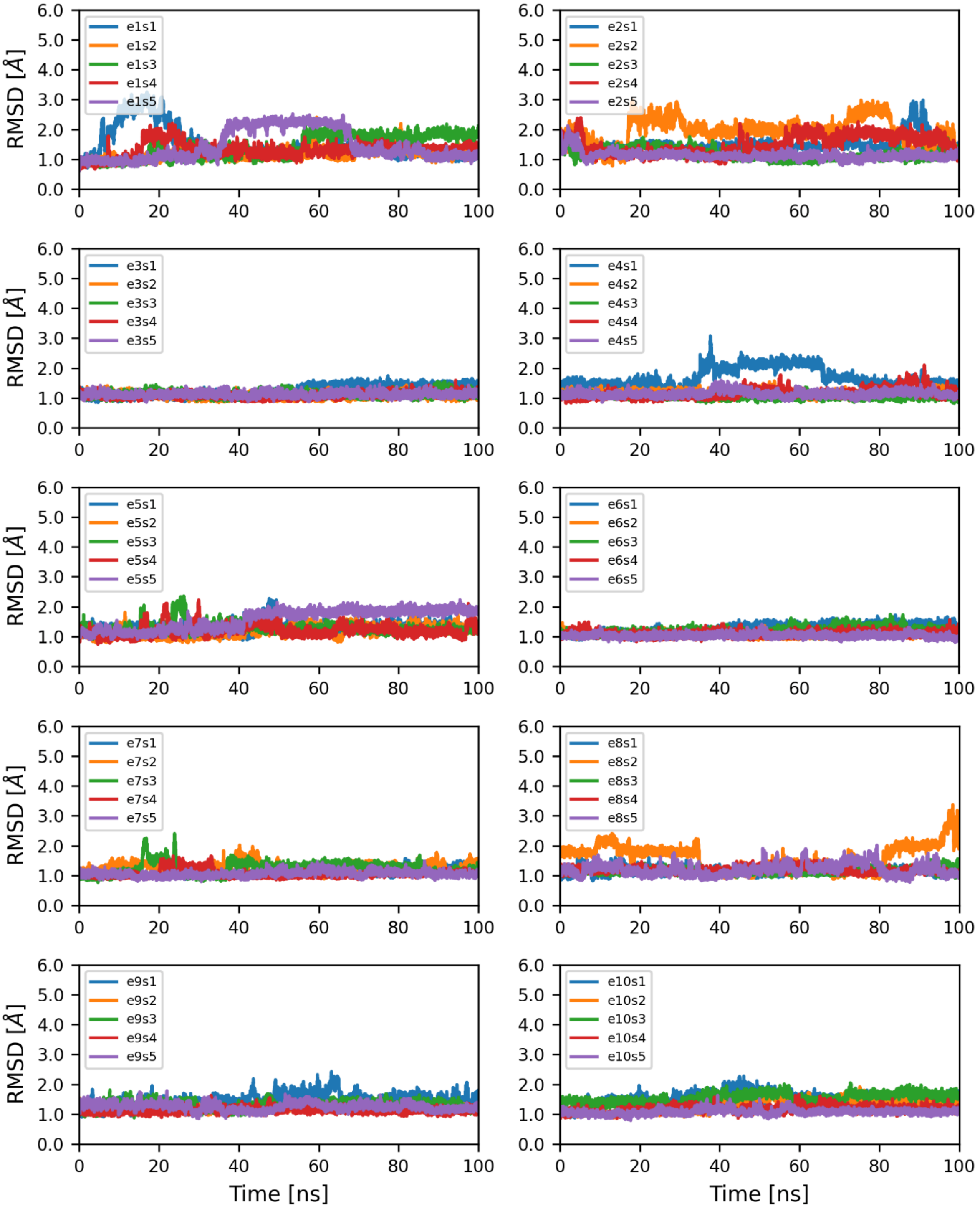

Figure S19. RMSD of Hal using TIP3P model for water molecules in adaptive MD simulations. 50 MD simulations of Hal at lower temperature 300 K grouped by epochs.

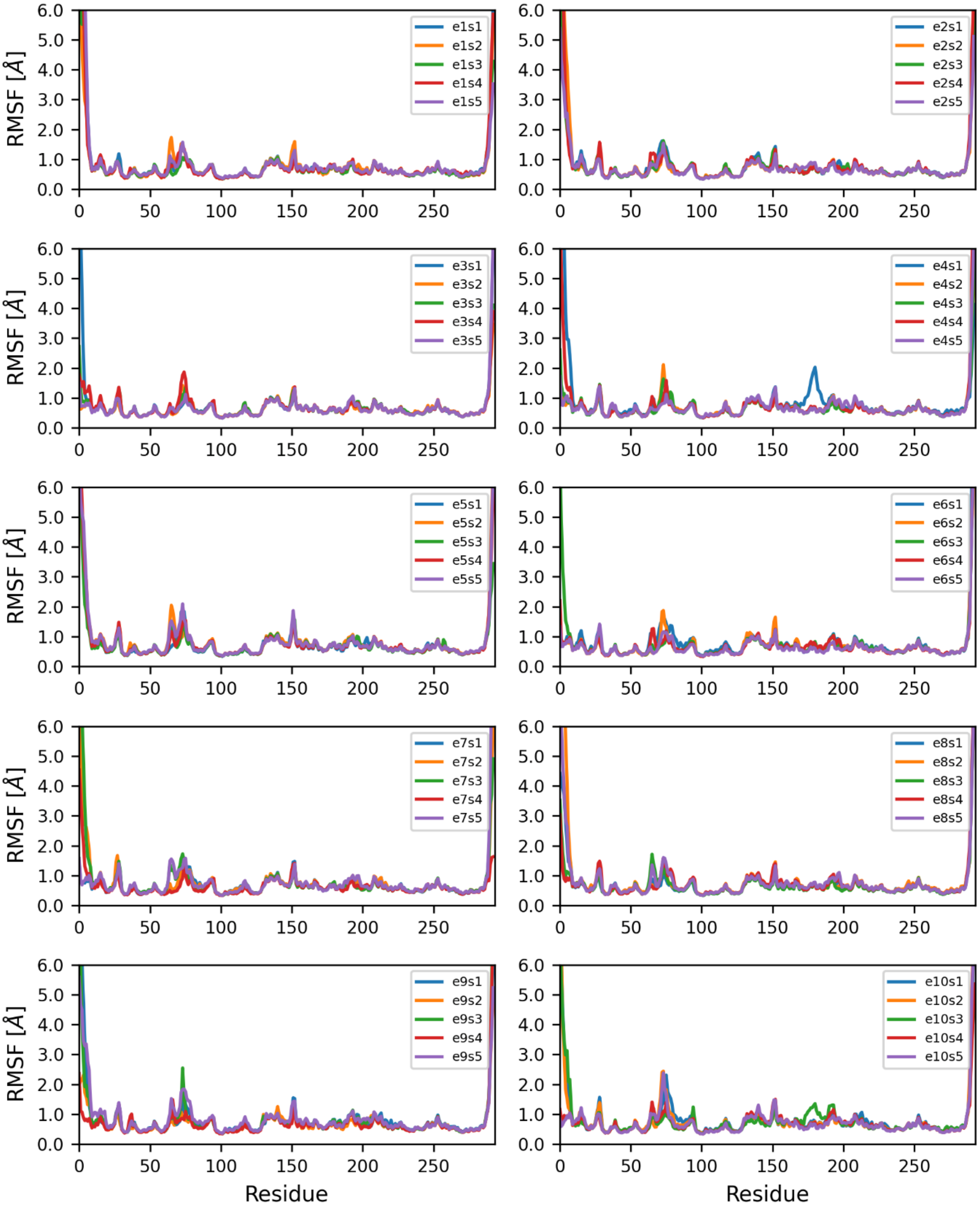

Figure S20. RMSF of Hal using TIP3P model for water molecules in adaptive MD simulations. 50 MD simulations of Hal at lower temperature 300 K grouped by epochs.

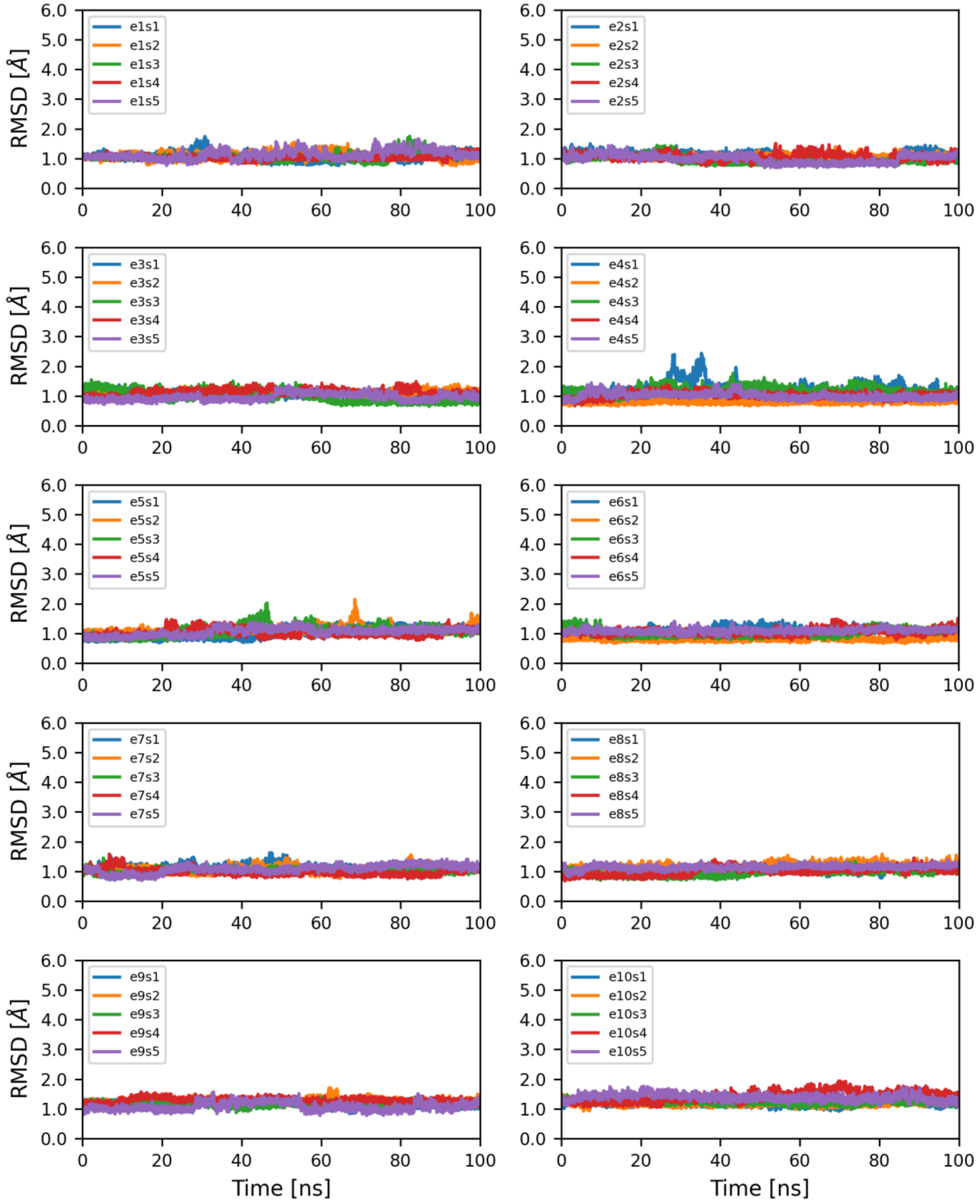

Figure S21. RMSD of Hal at the lower temperature of 300 K in adaptive MD simulations. 50 MD simulations of Hal using TIP3P model for water molecules grouped by epochs.

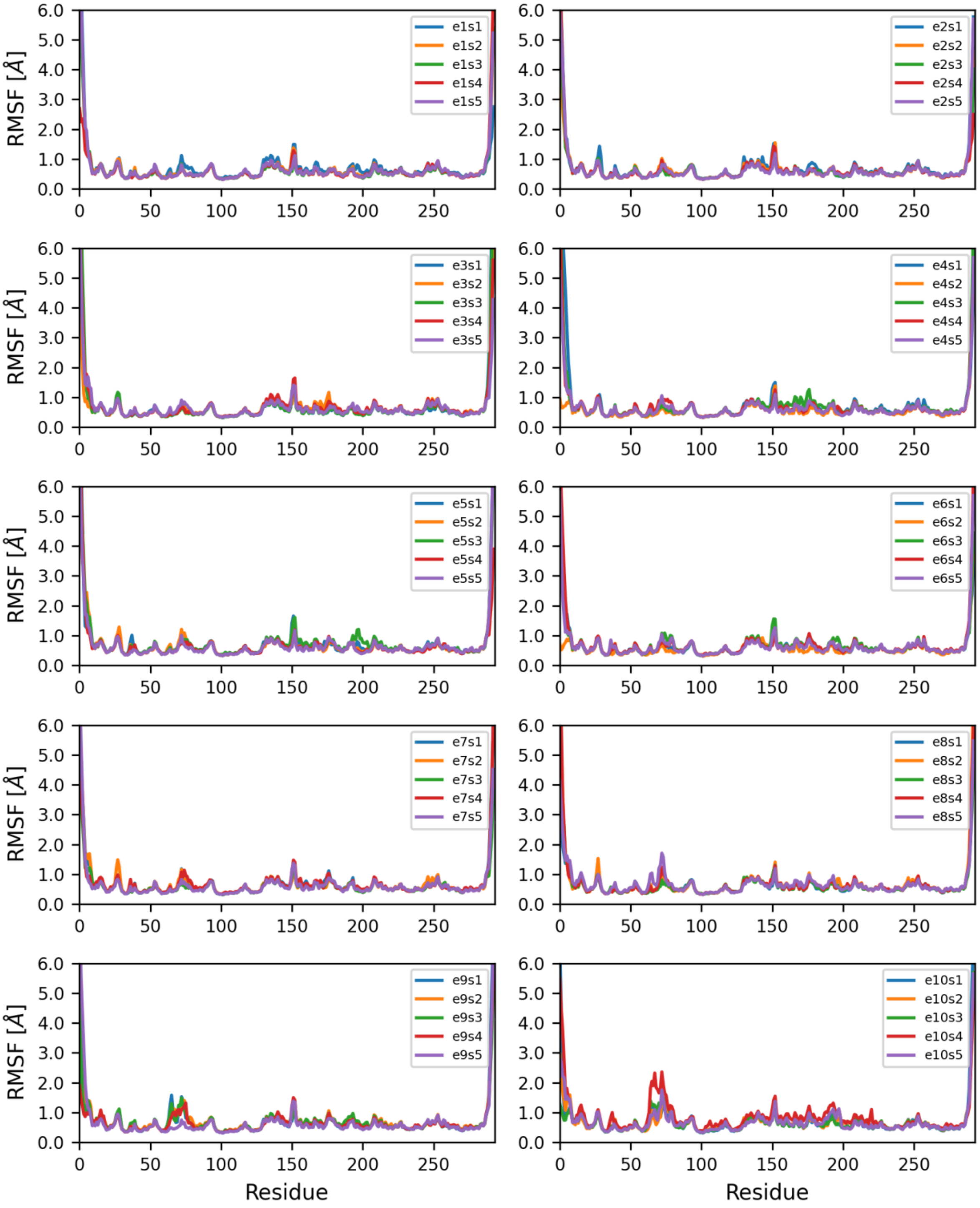

Figure S22. RMSF of Hal at the lower temperature of 300 K in adaptive MD simulations. 50 MD simulations of Hal using TIP3P model for water molecules grouped by epochs.

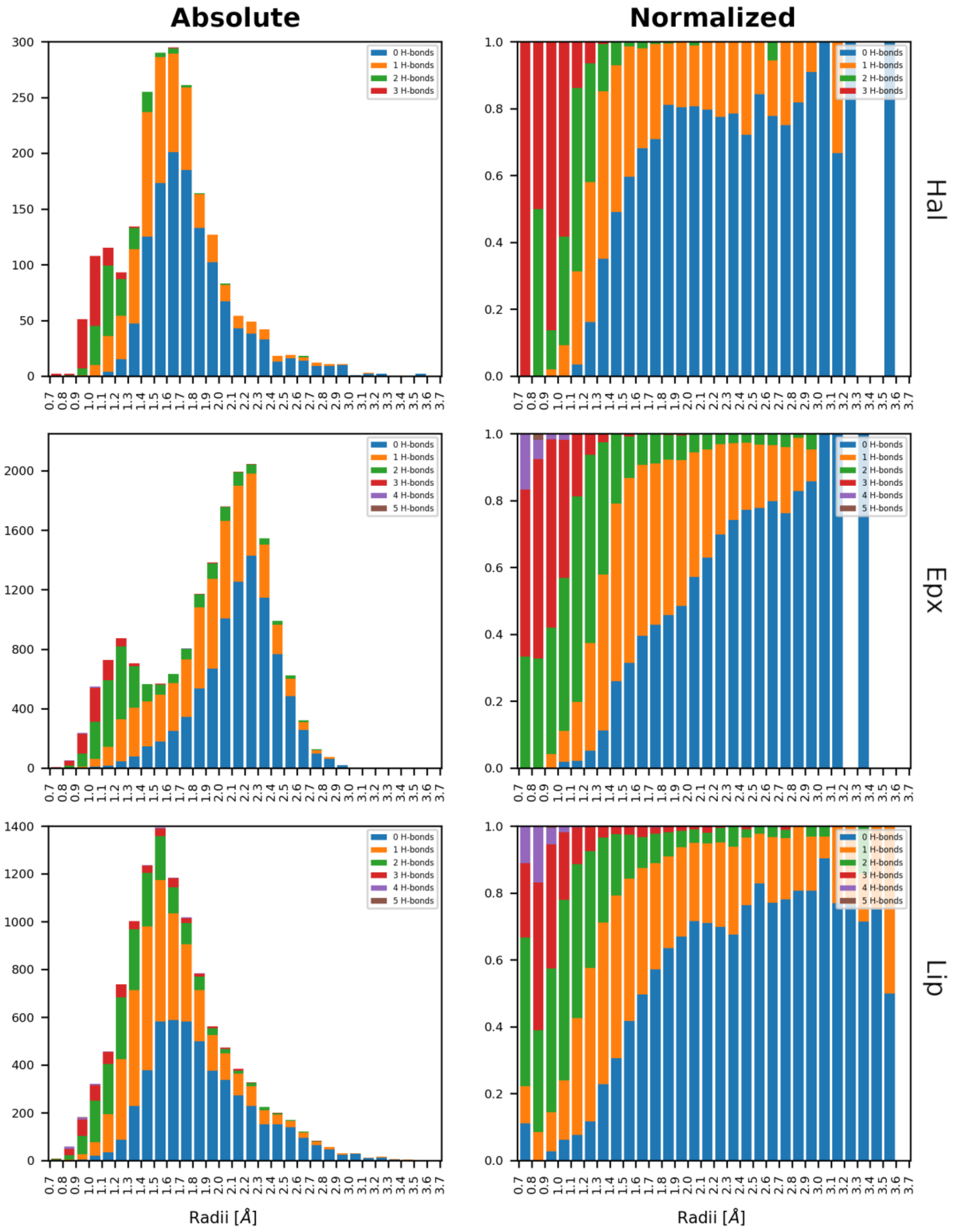

Figure S23. Distribution of *minimal event sphere* radii in Hal, Epx, and Lip by H-bonds. Histograms of the radius at which a water event occurs are presented in absolute and normalized scales. Colors represent the number of H-bonds present in each transport event.

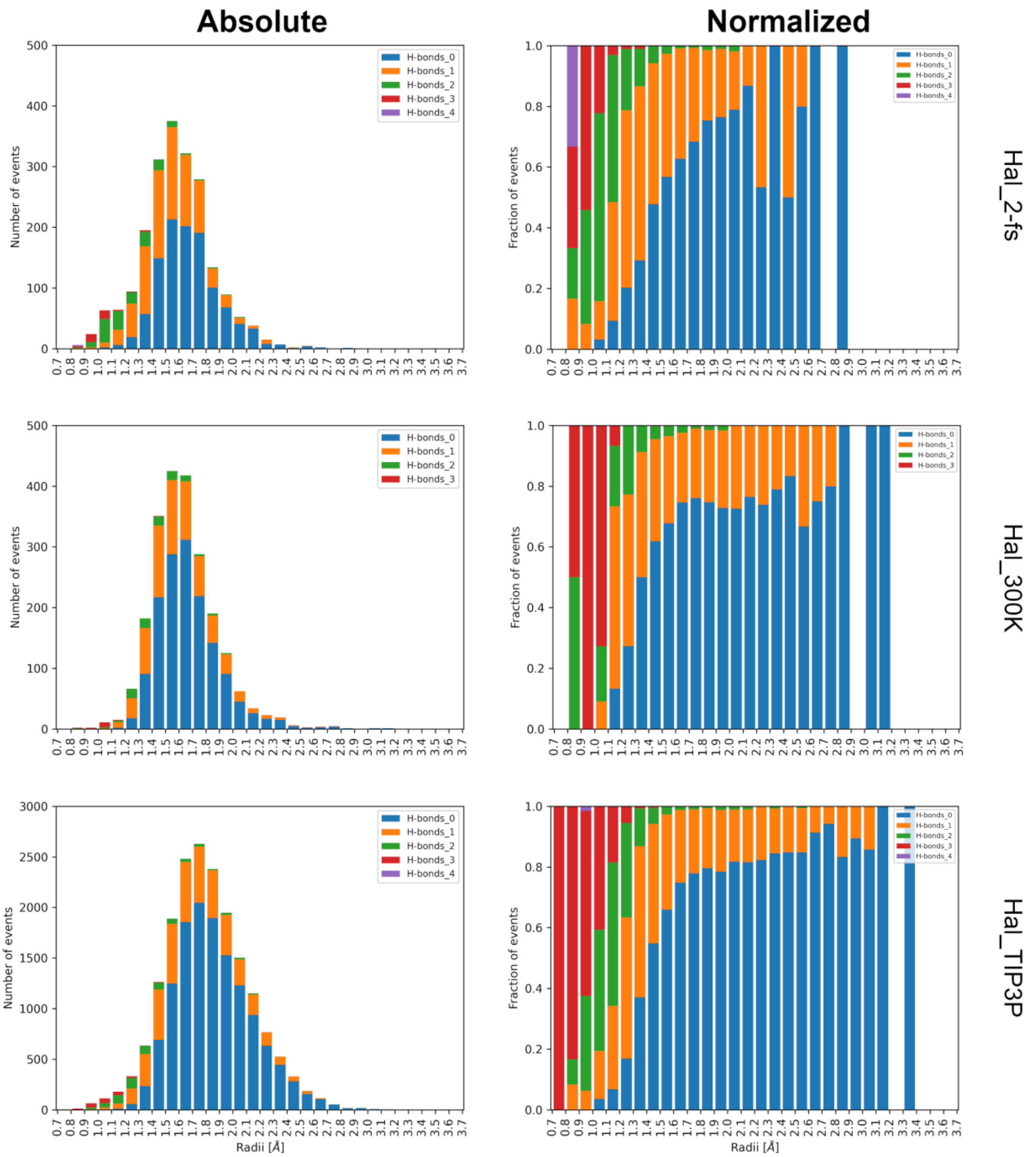

Figure S24. Distribution of *minimal event sphere* radii in Hal simulations under different setup by H-bonds. Histograms of the radius at which a water event occurs are presented in absolute and normalized scales for different simulation setups: i) simulations with 2-fs timestep and without HMR applied (Hal_2-fs), ii) simulations at lower temperature 300 K (Hal_300K), and iii) simulations using TIP3P model for water molecules (Hal_TIP3P). Colors represent the number of H-bonds present in each transport event.

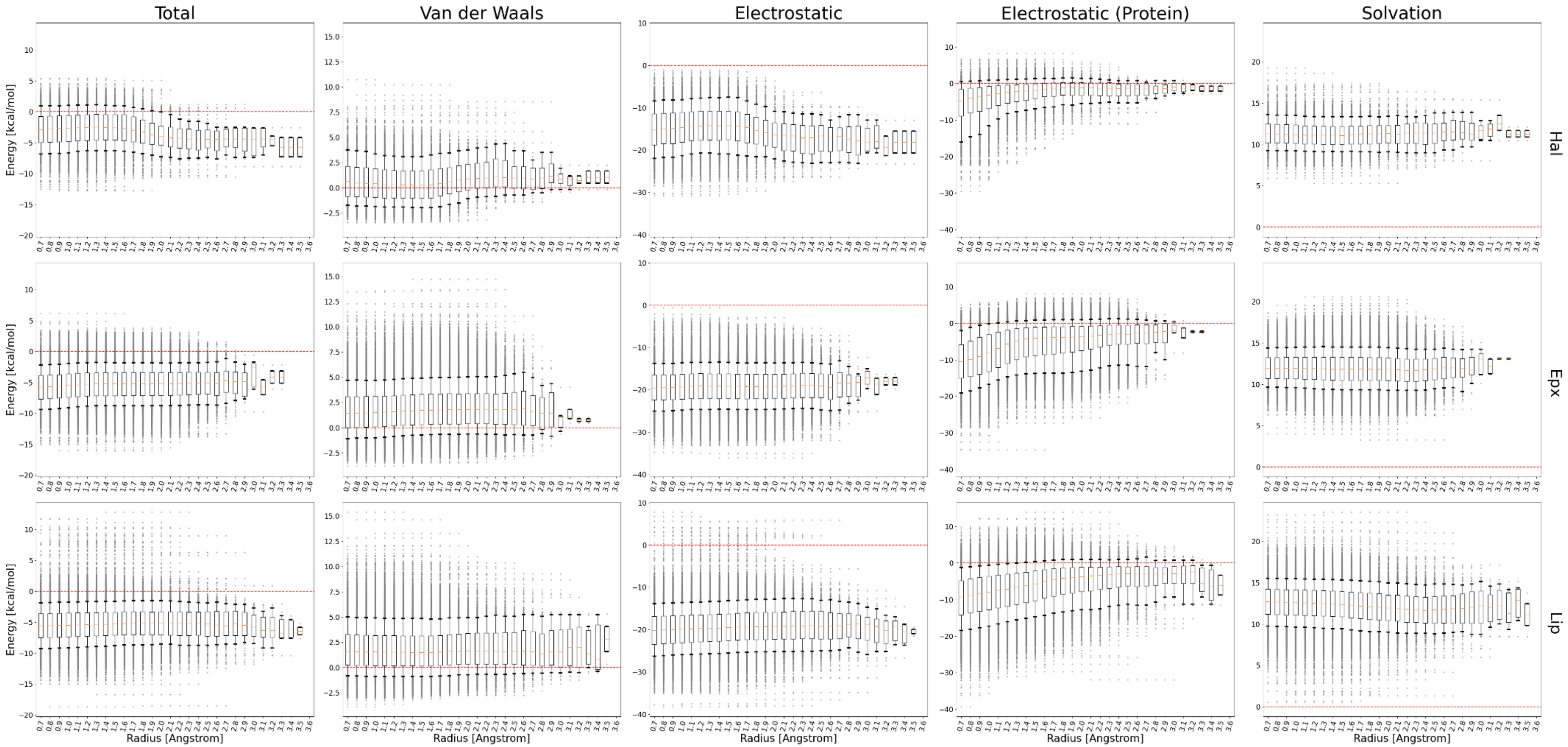

Figure S25. Binding free energy of water molecules present in individual bins of *minimal event sphere* radii in Hal, Epx, and Lip calculated by MMGBSA. The total binding energies as well as their contributing van der Waals, overall electrostatic, electrostatic with protein and solvation terms are reported. In each box-and-whiskers plot: the central orange line indicates the median; the box extends from the 25th to 75th percentile; the whiskers extend from the 10th to the 90th percentile, and outliers datapoint below and above the whiskers are marked by gray asterisks.

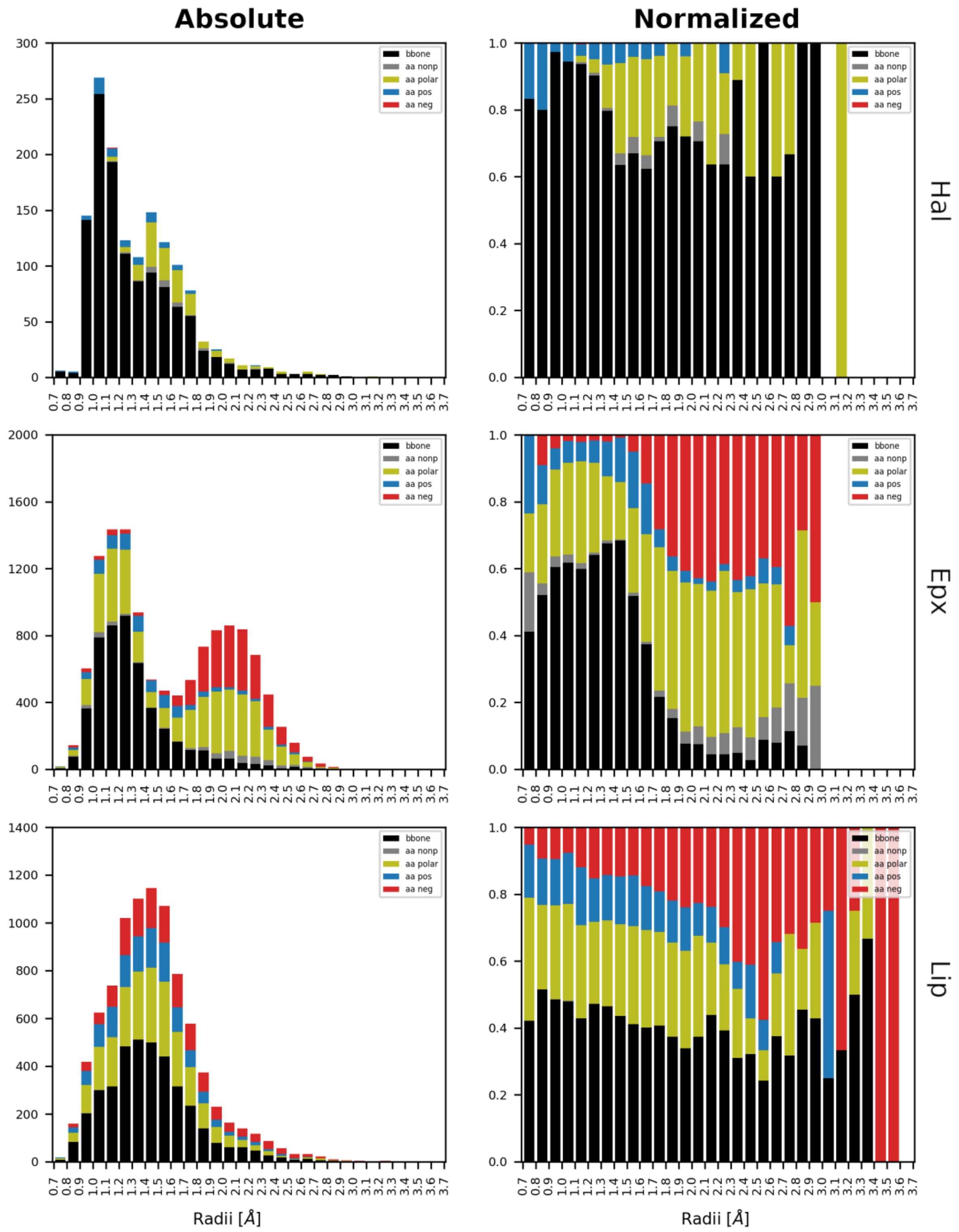

Figure S26. Distribution of atoms involved in H-bonds at *minimal event sphere* radii in Hal, Epx, and Lip. Histograms of the radius at which a water event occurs are presented in absolute and normalized scales for Hal, Epx, and Lip. Colors represent the type of atom involved in the H-bond present in each transport event.

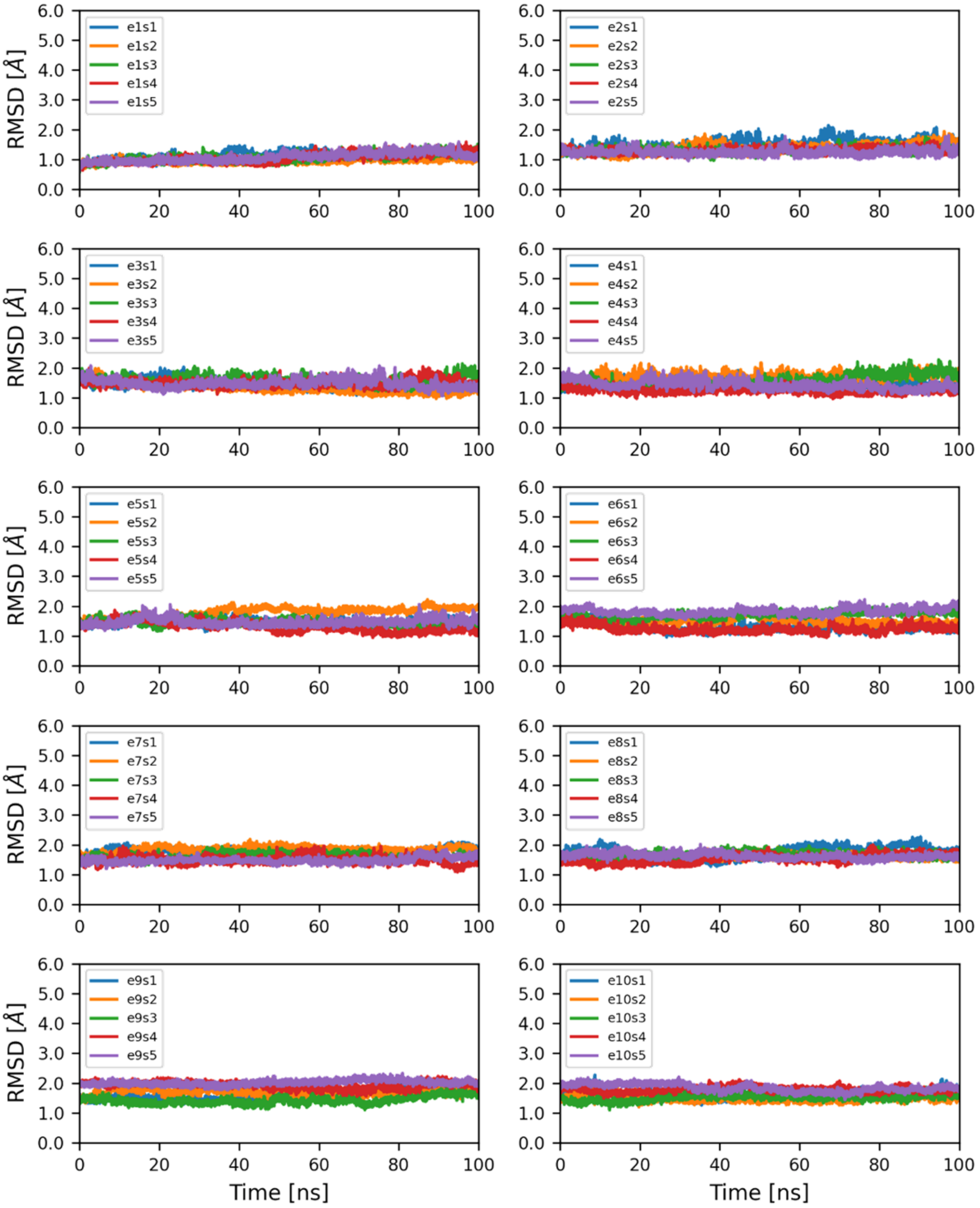

Figure S27. RMSD of hEpx in adaptive MD simulations. 50 MD simulations of hEpx grouped by epochs.

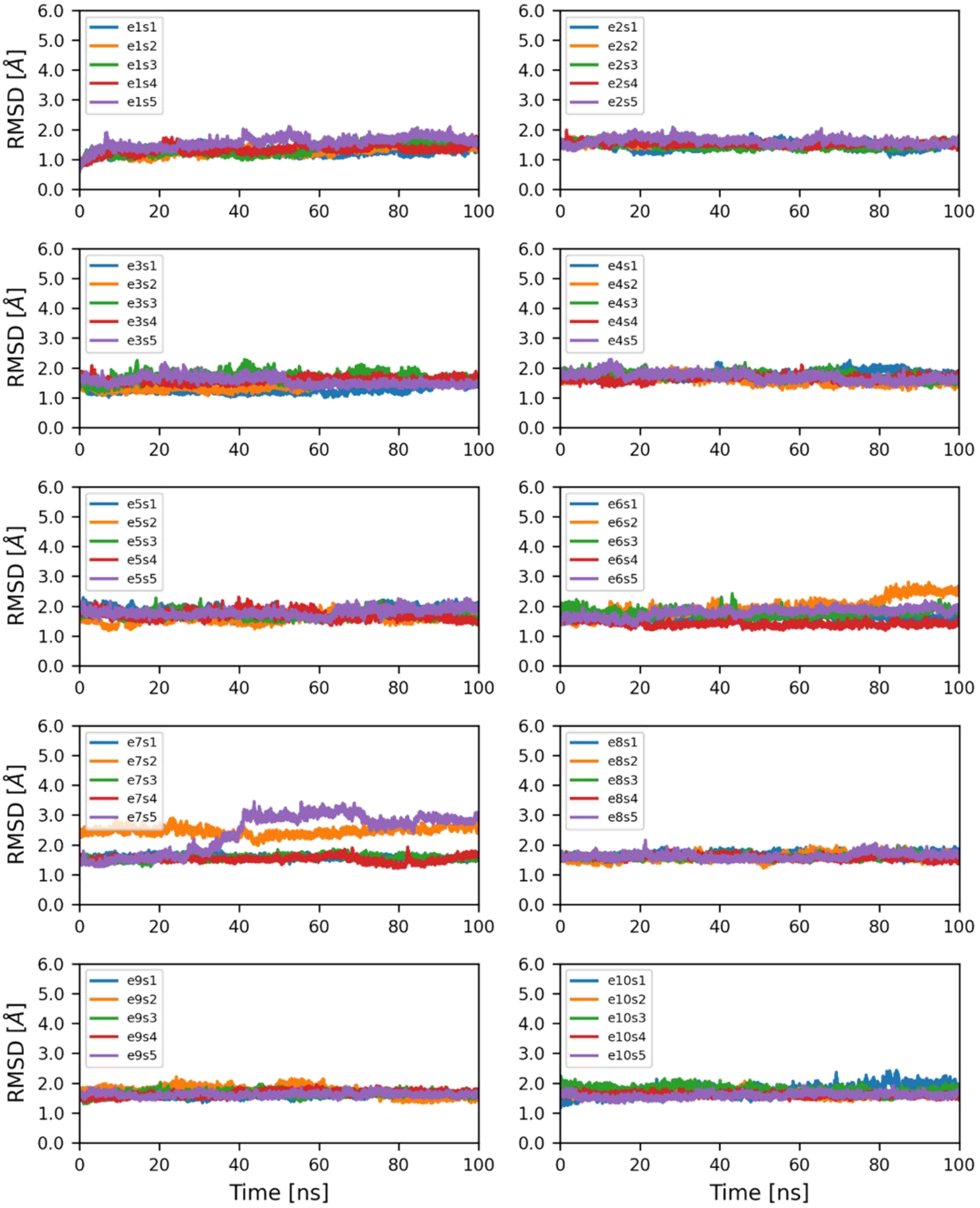

Figure S28. RMSD of E470G in adaptive MD simulations. 50 MD simulations of E470G grouped by epochs.

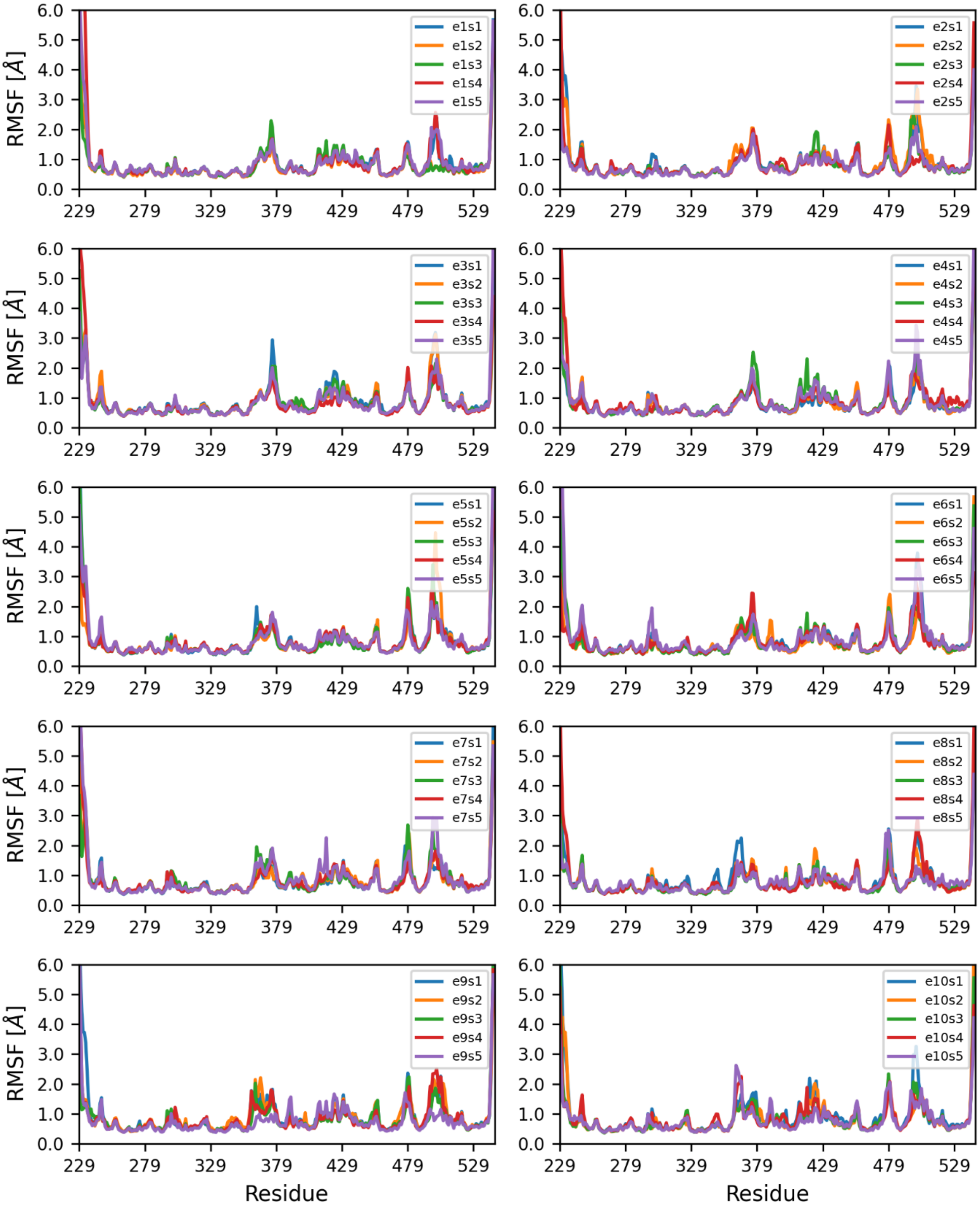

Figure S29. RMSF of hEpx in adaptive MD simulations. 50 MD simulations of hEpx grouped by epochs.

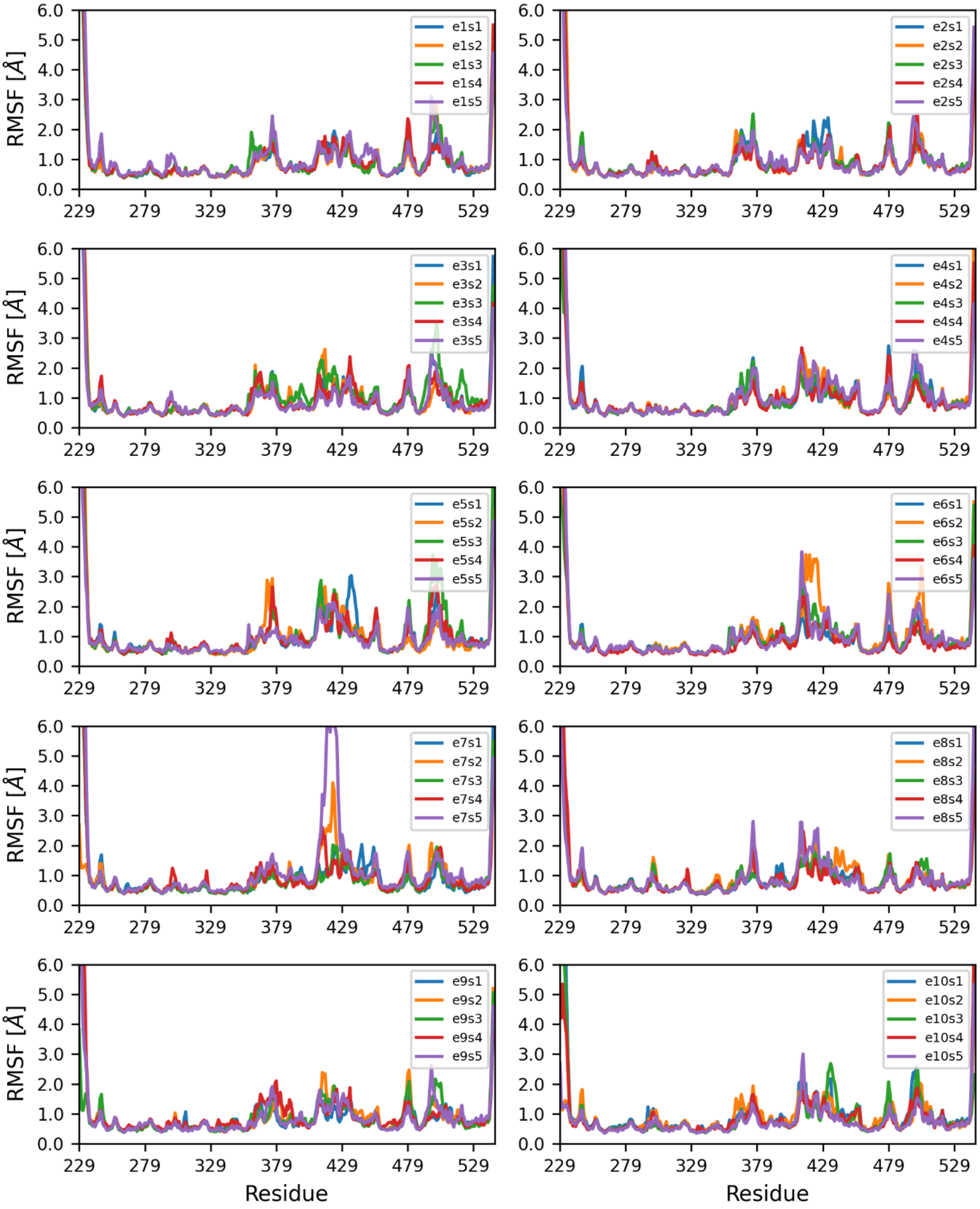

Figure S30. RMSF of E470G in adaptive MD simulations. 50 MD simulations of E470G grouped by epochs.

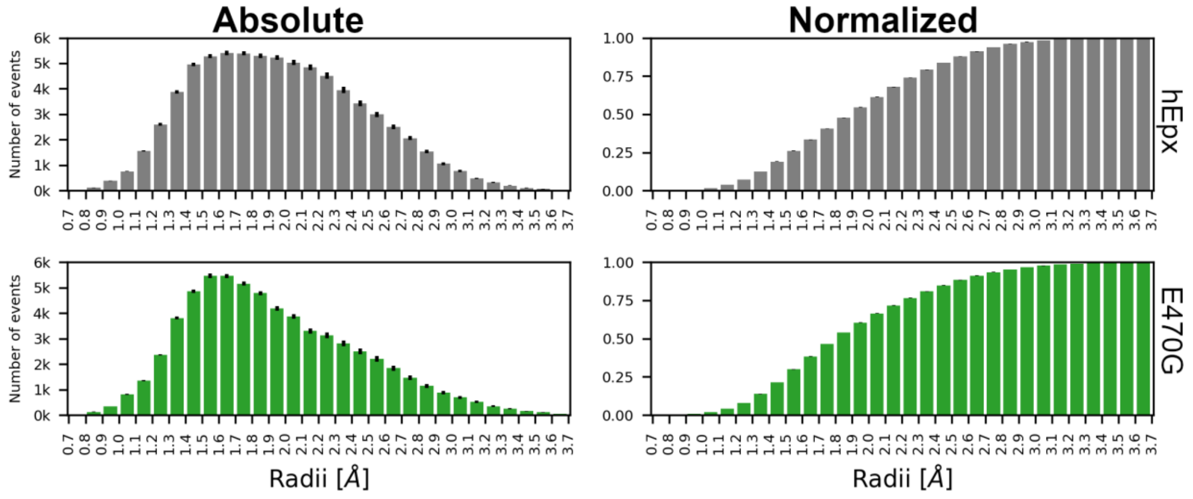

Figure S31. Distribution of *minimal event sphere* radii for water transport in hEpx and E470G. The absolute number of transport events and the corresponding normalized cumulative distribution, respectively. The variances were estimated by resampling 100 times 45 out of 50 available MD simulations; the number of simulations contributing to each bin is shown in **Figure S32**.

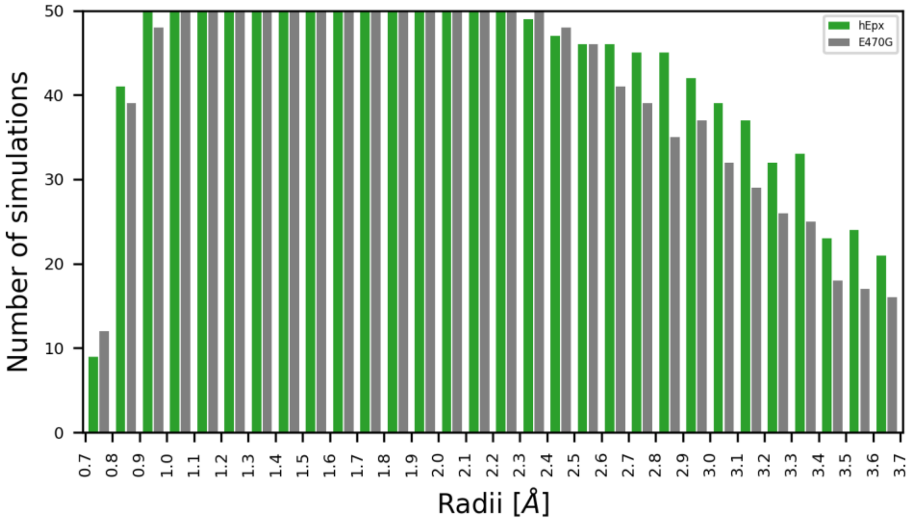

Figure S32. Number of MD simulations contributing events to each histogram bin in hEpx and E470G.

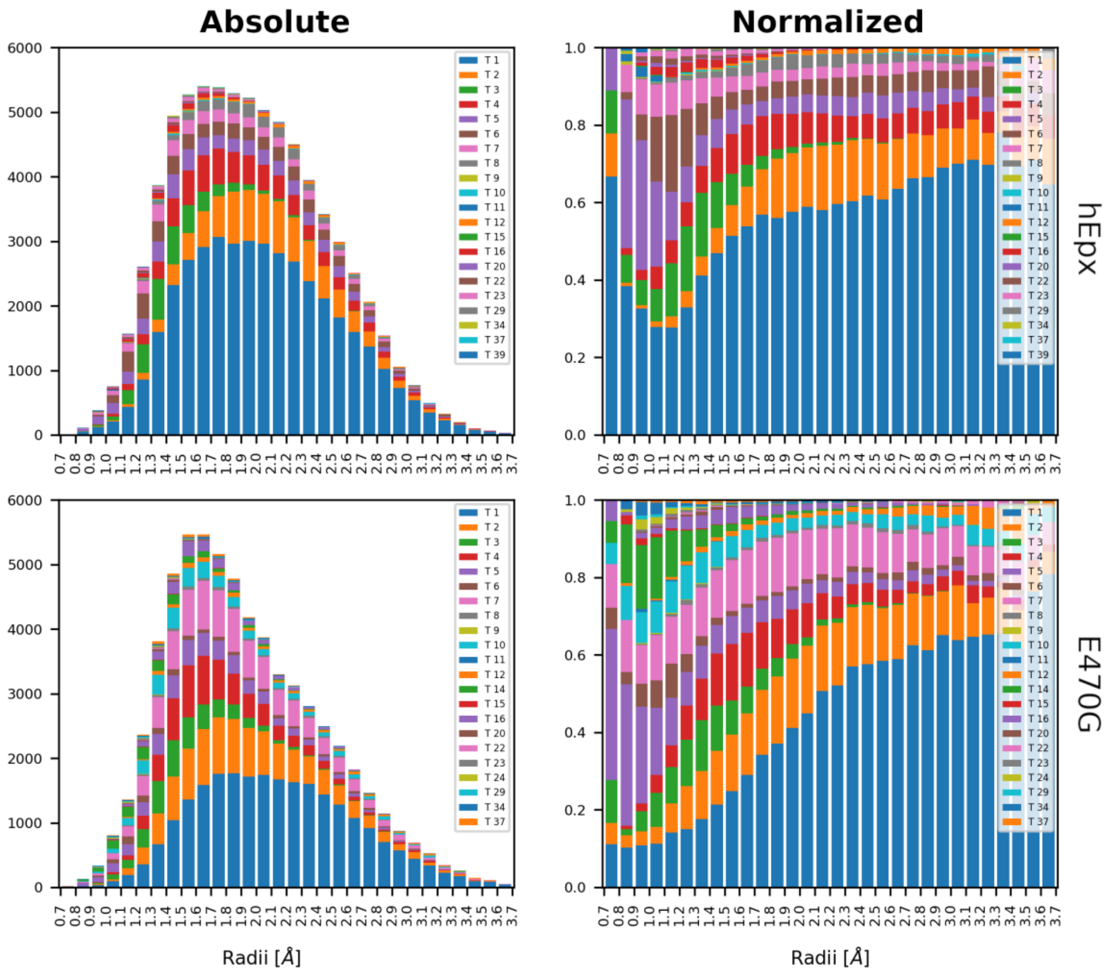

Figure S33. Distribution of *minimal event sphere* radii in hEpx and E470G by tunnels. Histograms of the radius at which a water event occurs are presented in absolute and normalized scales for hEpx and E470G. Colors represent individual clusters. However, it loops in due to the high number of tunnels identified.

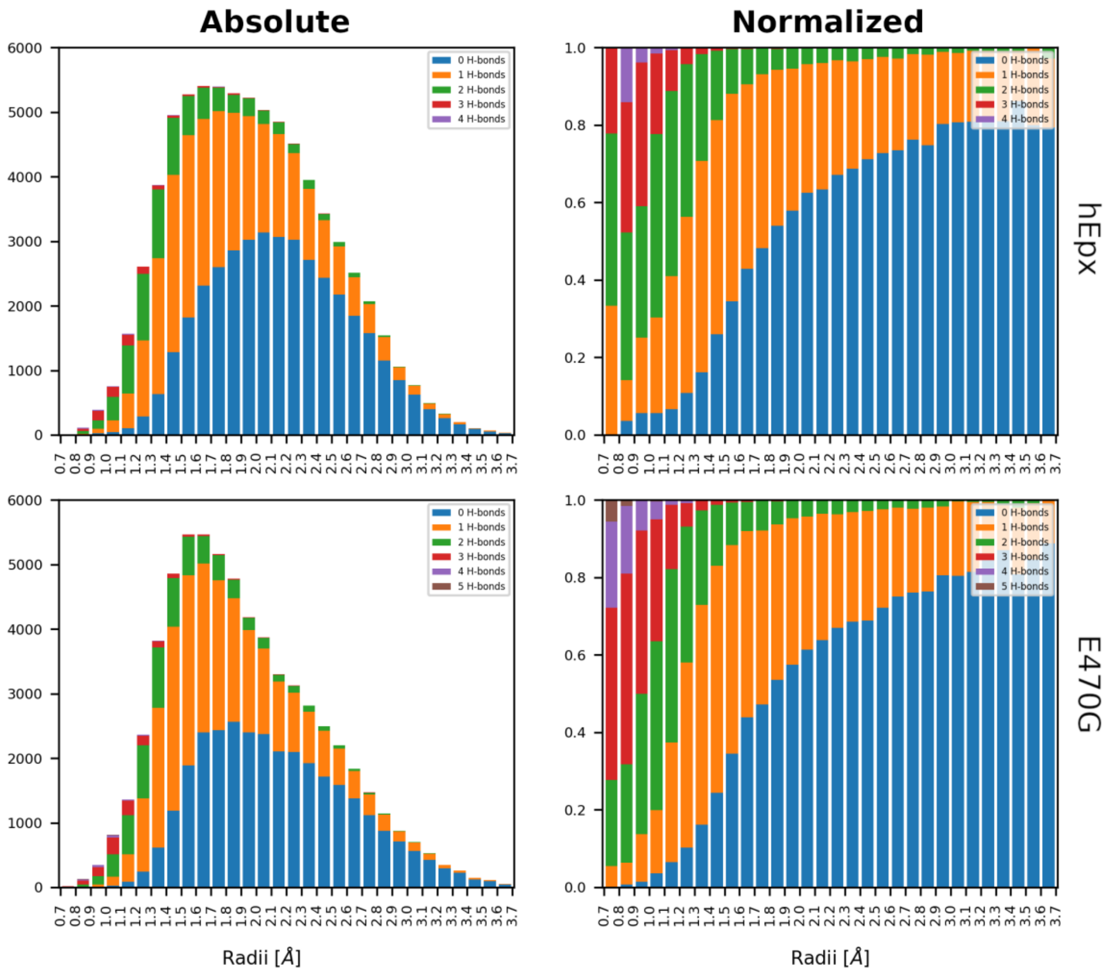

Figure S34. Distribution of *minimal event sphere* radii in hEpx and E470G by H-bonds. Histograms of the radius at which a water event occurs are presented in absolute and normalized scales for hEpx and E470G. Colors represent the number of H-bonds present in each transport event.

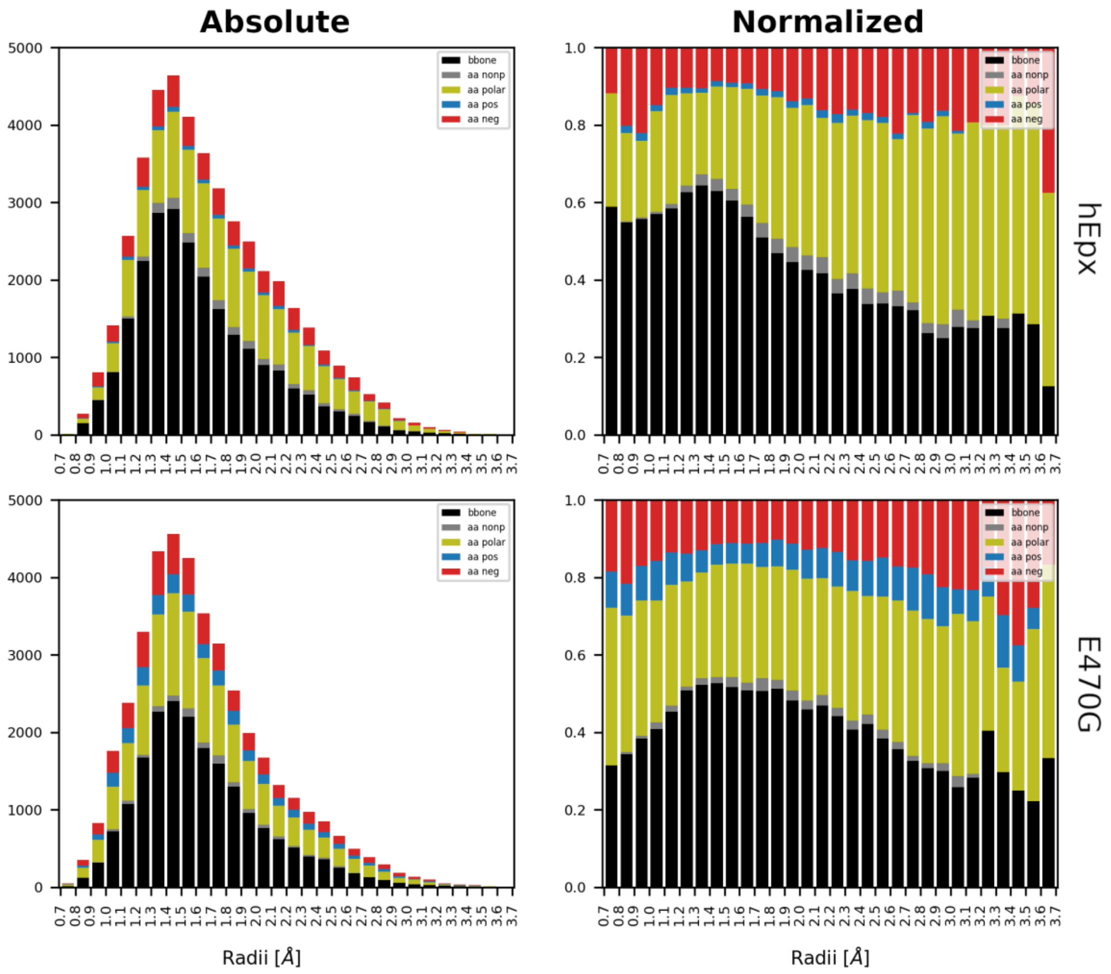

Figure S35. Distribution of atoms involved in H-bonds at *minimal event sphere* radii in hEpx and E470G. Histograms of the radius at which a water event occurs are presented in absolute and normalized scales for hEpx and E470G. Colors represent the type of atom involved in the H-bond present in each transport event.

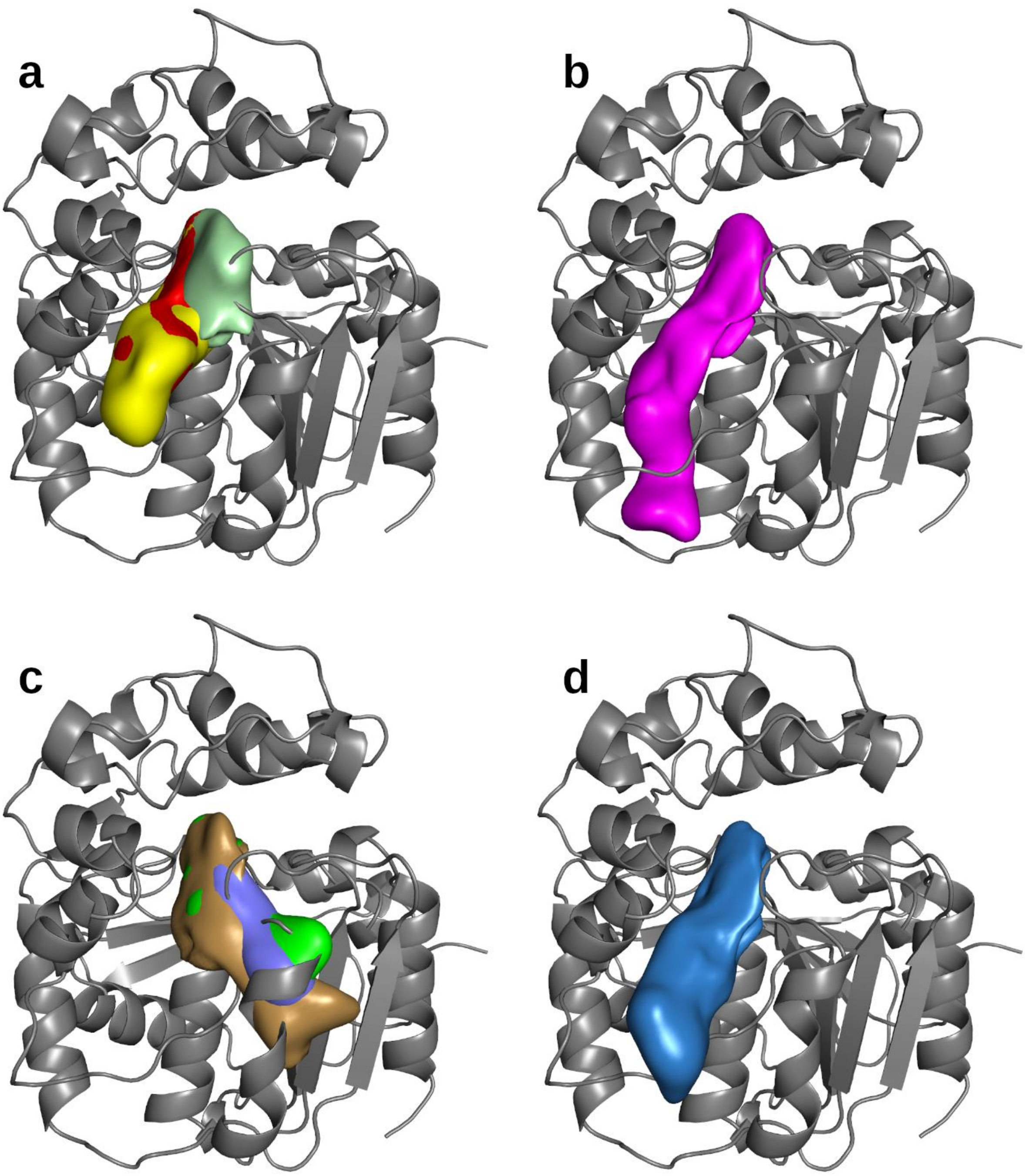

Figure S36. Tunnels identified in hEpx and E470G. Tunnels' densities at 20% of presence in hEpx and E470G obtained from 5 µs adaptive MD simulations. Tunnels described in the literature: a) *Tm1* (red, yellow, and pale green); b) *Tm5* (magenta); c) *Tm3* (sand, violet, and green); and d) *Tm2* (blue).

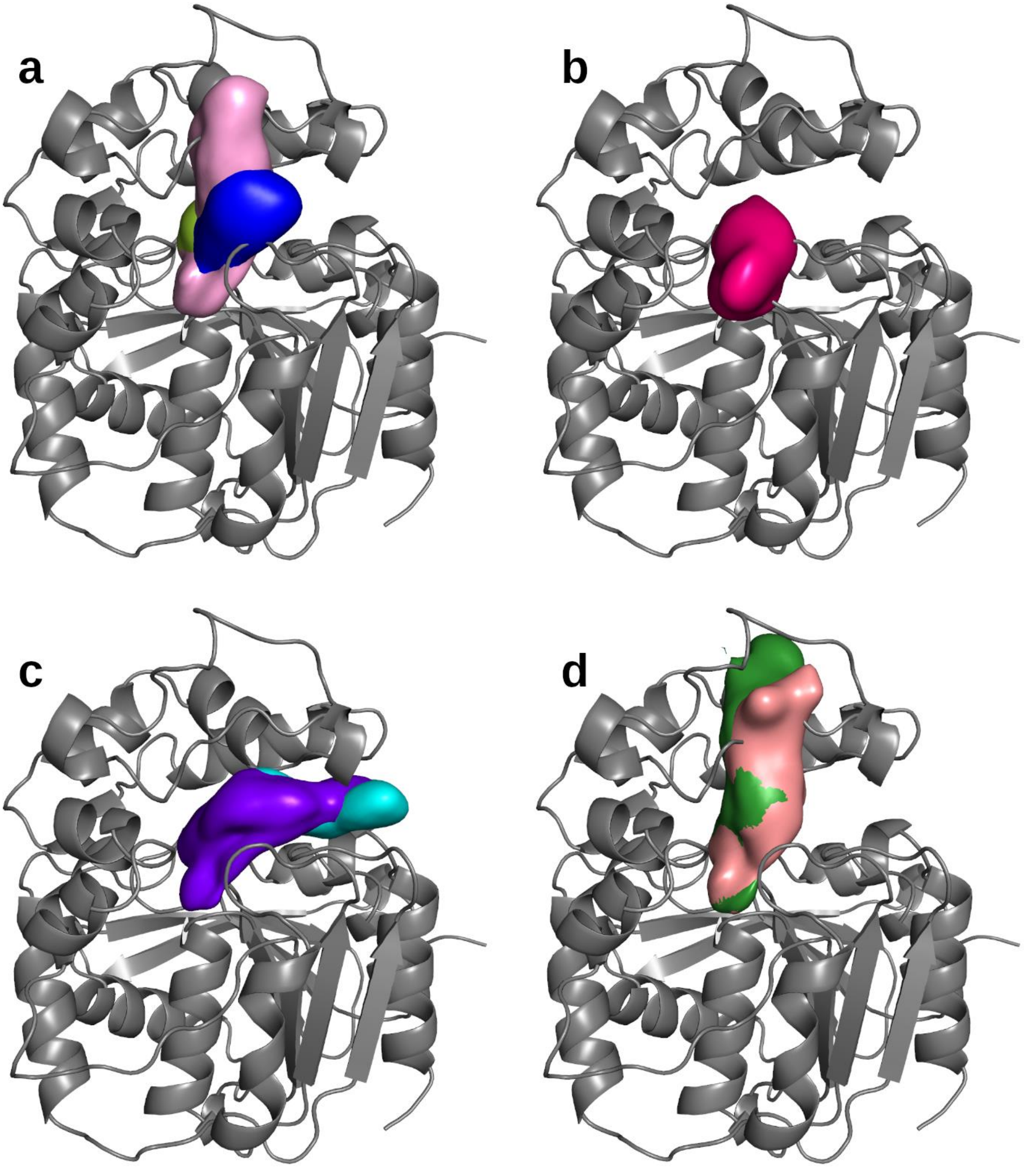

Figure S37. Tunnels identified in hEpx and E470G. Tunnels' densities at 20% of presence in hEpx and E470G obtained from 5 µs adaptive MD simulations. Tunnels described in the literature: a) *Tc/m* (blue, lemon, and pink); b)*Tg*; c) Tside (cyan and purple); and d) *Tcap1* (salmon and green).

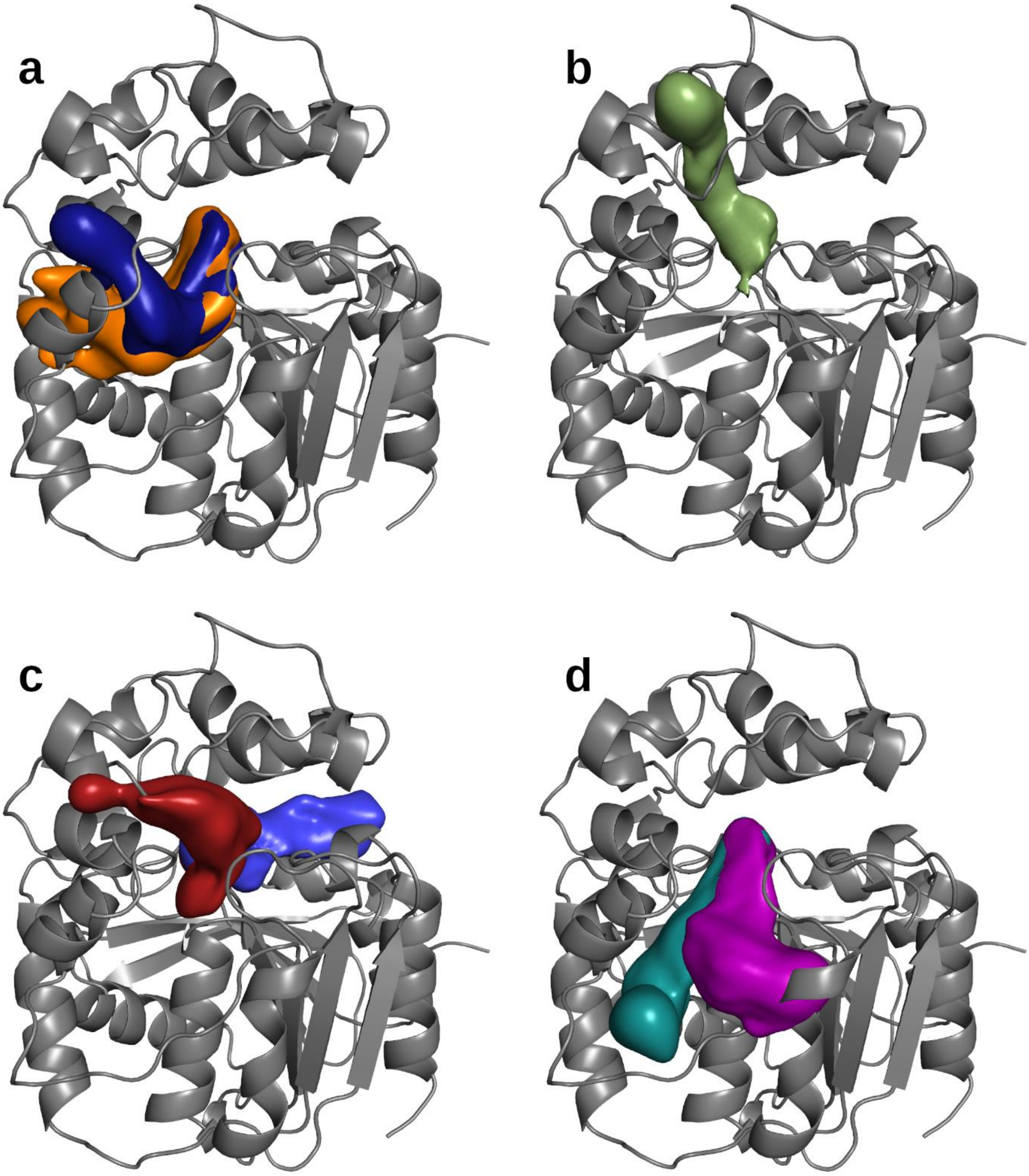

Figure S38. Tunnels identified in hEpx and E470G. Tunnels' densities at 20% of presence in hEpx and E470G obtained from 5 µs adaptive MD simulations. Tunnels described in the literature: a) *Tcap4* (orange and dark blue), b) *Tcap2*. Newly observed lesser tunnels that also participate in water transport c) *Tnew_A* and *Tnew_B* (in wine and blue, respectively) and d) *Tnew_C* and *Tnew_D* (in magenta and teal, respectively).

Table S1. Tunnels identified in Hal, Epx, and Lip and their corresponding naming in the literature.

Protein	Tunnel	Tunnel ID	Opening frequency	Average tunnel radii [Å]	Maximal tunnel radii [Å]	Transported molecules
**Hal**	*p1*	1	0.958	1.1±0.2	3.0	2080
	p2a	2	0.824	0.9±0.1	1.7	240
	p2b	30	0.005	0.9±0.2	1.8	6
	p2c	5	0.287	1.0±0.2	1.6	230
	p3	3	0.311	0.79±0.08	1.4	15
	side	7	0.169	0.78±0.08	1.5	7
	up	10	0.067	0.8±0.0.2	2.7	27
**Epx**	*TM1*	1, 5	1.000	1.9±0.3	3.3	49759
	TC/M	2	0.927	1.2±0.2	2.6	2399
	TM2	3	0.856	1.0±0.3	2.5	1907
	new_A	4	0.863	1.0±0.2	2.6	714
	new_B	6	0.568	0.9±0.1	1.9	16
	new_C	7	0.612	0.8±0.1	1.5	11
	new_D	8	0.4113	0.80±0.09	1.8	6
	new_E	9	0.475	0.8±0.1	1.9	11
	new_F	10	0.389	0.8±0.1	2.1	50
	new_G	11	0.382	0.8±0.1	2.5	42
	new_H	12	0.318	0.8±0.1	1.9	39
	new_I	13	0.439	0.8±0.1	1.7	7
	new_J	14	0.317	0.8±0.1	2.1	24
	new_K	15	0.346	0.8±0.1	1.6	32
	new_L	16	0.235	0.79±0.09	1.8	6
	new_M	17	0.294	0.8±0.1	1.6	8
	new_N	19	0.235	0.8±0.1	2.0	35
	new_O	21	0.223	0.77±0.08	1.5	27
	new_P	22	0.189	0.76±0.07	2.0	145
	new_Q	23	0.226	0.8±0.1	1.8	15
	new_R	24	0.200	0.8±0.1	1.7	10
	new_S	25	0.184	0.79±0.09	1.8	25
**Lip**	*main*	1, 4	1.000	1.8±0.4	3.7	32578
	ester	7	0.394	0.77±0.07	1.5	2
	new_A	2	0.573	1.2±0.4	3.0	3448
	new_B	3	0.556	1.0±0.3	2.6	2616
	new_C	5	0.684	1.0±0.2	2.1	266
	new_D	6	0.795	0.9±0.1	1.6	89
	new_E	8	0.542	0.8±0.1	2.0	143
	new_F	9	0.333	0.8±0.1	1.7	152
	new_G	10	0.368	0.8±0.1	1.7	77
	new_H	11	0.233	0.9±0.3	3.0	2097
	new_I	12	0.224	1.0±0.4	3.5	4136
	new_J	14	0.270	1.0±0.3	2.3	417
	new_K	15	0.339	0.8±0.1	1.9	44
	new_L	16	0.175	1.0±0.3	2.8	945
	new_M	17	0.246	0.8±0.1	1.6	9
	new_N	18	0.156	1.0±0.3	2.4	62
	new_O	19	0.212	0.8±0.1	1.7	50
	new_P	24	0.161	0.76±0.07	1.6	6
	new_Q	26	0.098	1.0±0.3	2.4	207
	new_R	29	0.076	0.9±0.2	2.0	51
	new_S	38	0.062	0.9±0.2	2.1	3
	new_T	66	0.006	1.4±0.3	2.3	5
	new_U	70	0.006	1.3±0.4	2.5	14

The major water transport tunnels are highlighted in dark gray, while the auxiliary tunnels are in light gray.

Table S2. Water events detected in Hal, Epx, and Lip and assigned to their tunnel networks.

Protein	Number of transport events		
	**Total**	**Assigned to TransportTools superclusters**	**Matched to a particular tunnel in the same MD simulation**
Hal	2658	2620	2222
Epx	55395	55336	17786
Lip	49192	47493	11086

Table S3. Water usage and average bottlenecks of tunnels from hEpx and E470G.

Tunnel	Water usage			Average Bottleneck Radius		
	**WT**	**E470G**	**Difference**	**WT**	**E470G**	**Difference**
**1**	41543	25008	**16535**	1.904	1.836	0.068
2	9033	9482	-449	1.676	1.689	-0.013
3	3265	3309	-44	1.223	1.304	-0.081
4	5866	5684	182	1.443	1.460	-0.017
5	4169	3655	514	1.089	1.309	-0.220
**6**	3846	1060	**2786**	1.074	0.989	0.085
**7**	2653	7548	**-4895**	1.221	1.515	-0.294
**8**	1820	515	**1305**	1.663	1.652	0.011
9	22	26	-4	0.793	0.797	-0.004
**10**	160	2687	**-2527**	0.954	1.082	-0.128
11	26	15	11	0.818	0.777	0.041
12	571	898	-327	1.270	1.191	0.079
**14**	0	1343	**-1343**	0.820	1.020	-0.200
15	11	61	-50	0.851	0.872	-0.021
16	622	1572	-950	1.097	1.259	-0.162
20	152	182	-30	0.923	0.938	-0.015
22	213	104	109	1.093	0.975	0.118
23	462	121	341	1.233	1.117	0.116
24	0	113	-113	0.742	0.884	-0.142
29	132	60	72	1.539	1.767	-0.228
34	81	316	-235	1.153	1.108	0.045
37	63	288	-225	1.408	1.369	0.039
39	44	0	44	0.842	0.791	0.051
**Total**	**74754**	**64047**	**10707**			
